# Metformin does not slow cyst growth in the PCK rat model of polycystic kidney disease

**DOI:** 10.14814/phy2.15776

**Published:** 2023-08-31

**Authors:** Ozgur A. Oto, Daniel J. Atwood, Anjana Chaudhary, Zhibin He, Amy S. Li, Michael F. Wempe, Charles L. Edelstein

**Affiliations:** ^1^ Division of Renal Diseases and Hypertension University of Colorado Anschutz Medical Campus Aurora Colorado USA; ^2^ Department of Pharmaceutical Sciences, Skaggs School of Pharmacy and Pharmaceutical Sciences University of Colorado Anschutz Medical Campus Aurora Colorado USA

## Abstract

Metformin (MET) has the potential to activate p‐AMPK and block mTORC1‐induced proliferation of tubular cells in PKD kidneys. The aim of this study was to determine the effects of MET on cyst growth, kidney function, AMPK and mTOR signaling, and lactate levels in male PCK rats, a Pkhd1 gene mutation model of human autosomal recessive polycystic kidney disease (ARPKD). MET 300 mg/kg/day IP from days 28 to 84 of age resulted in a mean serum metformin level that was 10 times the upper limit of therapeutic, no effect on cyst indices, nephrotoxicity, and increased serum lactate. MET 150 mg/kg resulted in a therapeutic serum metformin level but had no effect on kidney weight, cyst indices, kidney function, or mTOR and autophagy proteins. In summary, a standard dose of MET was ineffective in reducing PKD, did not activate p‐AMPK or suppress mTOR and the higher dose resulted in increased lactate levels and nephrotoxicity. In conclusion, the study dampens enthusiasm for human studies of MET in PKD. Doubling the metformin dose resulted in a 10‐fold increase in mean blood levels and toxicity suggesting that the dosage range between therapeutic and toxic is narrow.

## Graphical abstract

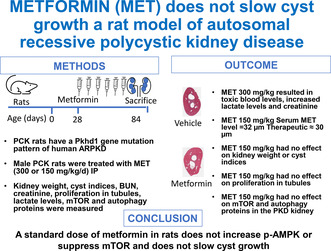

**New and Noteworthy**

- Metformin has not previously been tested in a rat model of polycystic kidney disease.
- Therapeutic blood levels of metformin did not slow PKD. Doubling the metformin dose resulted in a 10‐fold increase in mean blood levels and toxicity suggesting that the dosage range between therapeutic and toxic is narrow.
- Metformin did not activate p‐AMPK or inhibit proliferation in the PKD kidney.
- The study raised concerns about using metformin in patients with PKD.

## 1 INTRODUCTION

Autosomal‐dominant polycystic kidney disease (ADPKD) is the commonest inherited kidney disease and is characterized by the slow and persistent growth of renal tubular epithelial cell‐lined cysts. Over time, these fluid‐filled cysts change the gross anatomy of the kidney, normal parenchymal structures are compressed, and kidney function is impaired. Proliferation and fluid secretion of the cells lining the cyst are the major mechanisms of cyst growth (Belibi & Edelstein, 2010). Metformin (MET) increases AMPK which has the potential to inhibit mTORC1, a potent driver of proliferation of cells lining the cysts that is a universal mechanism of cyst growth. The effect of MET on cyst growth and kidney function has been tested in mouse and human PKD (Brosnahan et al., 2022; Chang et al., 2022; Leonhard et al., 2019; Pastor‐Soler et al., 2022; Perrone et al., 2021; Takiar et al., 2011). The effect of MET on PKD has not been studied in rat or ARPKD models. The PCK rat, a Pkhd1 gene mutation model of human ARPKD, that has many of the phenotypic features of human ADPKD, is a good model to further understand the beneficial and adverse effects of MET in PKD and to provide insights into designing human studies (Lager et al., 2001).

Preclinical and clinical studies of MET in PKD have shown mixed results. MET reduced cyst growth in vitro and in ex vivo kidney cyst models and in vivo in two rapid cyst growth models of PKD (Takiar et al., 2011). MET had no effect on cyst growth in an adult onset slowly progressive model of PKD more analogous to human disease than the rapid models (Leonhard et al., 2019). The effect of MET in the Pkd1^RC/RC^ mouse hypomorphic gene model of PKD is dependent on the background strain and rate of progression of PKD (Chang et al., 2022; Pastor‐Soler et al., 2022). In Pkd1 miRNA transgenic mice, MET had no effect on cyst growth (Chang et al., 2022). In a prospective randomized controlled double‐blind feasibility study, MET did not change kidney volume or kidney function in ADPKD patients (Brosnahan et al., 2022). In a randomized trial of MET in ADPKD patients (TAME PKD), the annual change of GFR did not reach statistical significance in the MET‐treated group (Perrone et al., 2021). However, it is concerning that only a few of the preclinical studies demonstrated that MET could increase AMPK, suppress mTORC1, and inhibit proliferation in vivo in the PKD kidney. It is possible that activation of AMPK, suppression of mTORC1, and inhibition of proliferation in the human PKD kidney may require a dose of MET that is potentially toxic.

The PCK rat is a clinically relevant model that has the same gene mutation of human ARPKD and has slowly progressive cystic kidney and liver disease that phenotypically resembles ADPKD (Lager et al., 2001). MET has not been tested in a rat model of PKD. The PCK rat offers a clinically relevant model to study the beneficial and adverse effects of MET on PKD and to determine whether standard doses of MET can activate AMPK, suppress mTORC1, activate autophagy, and inhibit proliferation in the kidney and protect against cyst growth.

## 2 METHODS

### 2.1 In vivo model

In this study, male PCK rats were used (Katholnig et al., 2013). PCK rat breeding pairs were obtained from Dr Peter Harris (Mayo Clinic College of Medicine). The PCK rats have a Pkhd1 gene mutation pattern of human ARPKD. In these rats, progressive renal cystic disease becomes evident from the first week of life. PCK rats have increased liver weight by 21 days of age and markedly enlarged liver by 180 days of age (Katholnig et al., 2013). The kidney cysts develop as a focal process from the loop of Henle, distal, and collecting tubules, especially localized in the corticomedullary region and outer medulla. PKD is more severe in males than in females, while the severity of the liver disease is not different in both sexes (Lager et al., 2001). Thus, in the present study, only male PCK rats were studied. All experiments were performed following the guidelines in the National Institutes of Health Guidelines for the Care and Use of Laboratory Animals. The Animal Care and Use Committee of the University of Colorado at Denver approved the animal protocol. Rats were placed on a standard diet under standard pathogen‐free housing conditions where food and water were freely available.

### 2.2 Experimental in vivo protocol

Animals were randomly assigned to treatment with MET (300 mg/kg/day or 150 mg/kg/day body weight) or vehicle (sterile saline) by intraperitoneal (IP) injection from 28 to 84 days of age. MET was administered intraperitoneally after dissolving with sterile saline. MET was obtained from Sigma‐Aldrich. At the end of the treatment period, rats were sacrificed under isoflurane anesthesia. Blood samples were obtained with a cardiac puncture and then the kidneys, heart, and liver were removed and weighed. Slices of organs were preserved in formaldehyde and embedded in paraffin for histological examination.

### 2.3 MET dosing

The no observable adverse effect level (NOAEL) of MET is 200 mg/kg/day orally in Sprague Dawley rats (Quaile et al., 2010). MET 200 mg/kg results in an area under the curve (AUC) 0–24 and C_max_ (sex averaged) of 41.1 μg h/mL and 10.3 μg/mL, respectively, which corresponds to ∼2× and 8× the human AUC and C_max_ following a 2000‐mg total daily dose (Timmins et al., 2005). MET IP which in general results in a lower blood level than oral administration in rodents was used (Chandel et al., 2016). Doses of 150–300 mg/kg IP that have shown a therapeutic effect in rat models of spinal cord injury (Wang et al., 2022) and stroke (Zemgulyte et al., 2021) were used.

### 2.4 Measurement of kidney function

Blood urea nitrogen was determined according to the manufacturer's instructions (DIUR‐100) using a urea assay kit (BioAssay Systems). Serum creatinine levels were measured by HPLC‐tandem mass spectrometry.

### 2.5 Immunoblot analysis

Proteins were isolated in tissues using RIPA, whole protease, and phoSTOP phosphatase inhibitor cocktails (Sigma) as we have previously described (Atwood, Brown, et al., 2020; Atwood, Pokhrel, et al., 2020). The obtained homogenates were centrifuged, and the supernatant was collected for protein quantification with BioRad DC protein assay, following the manufacturer's instructions. Samples were mixed with Laemmli sample buffer and boiled for 5 min. The samples were transferred to 0.45 μm PVDF membranes after running on 4%–20% precast polyacrylamide gels. These membranes were then blocked with 2.5% evaporated milk and probed with the following antibodies at a dilution of 1:1000 obtained from Cellular Signaling Technology, Danvers, MA: AKT # 9272, p‐Akt^S473^ # 9271, p‐Akt^S308^ # 9275, AMPK # 2532, p‐AMPK^T172^ # 2535, p‐ACC ^S79^ # 3661, ACC #3662, GAPDH # 2118, LC3‐II # 2775, mTOR # 2983, p62 # 5114, S6 # 2217, pS6^S235/236^ # 2211, and anti‐rabbit IgG HRP # 7074. The specificity of each of these antibodies used has been validated by the vendor (Cell Signaling Technology). Blots were developed with chemiluminescence, and densitometric analysis was performed using ImageJ.

The molecular weight of protein bands was confirmed by adding 5 μL of PageRulerTM Prestained Protein Ladder (Thermofisher) to the first and last well of the SDS‐polyacrylamide gel used for the immunoblot. This ladder was visible to the naked eye after being transferred to a nitrocellulose membrane. The film was placed in a dark cassette firmly aligned to one side of the cassette to expose the film to fluorescing protein bands. After the desired exposure time, the film was developed and the film was placed back into the cassette in the original alignment. The visible molecular weight ladders framing the nitrocellulose membrane were then traced onto the film with a black marking pen (Figure S1).

### 2.6 Routine histology

Hematoxylin–eosin‐stained kidneys were scanned using an Aperio ImageScope (Leica Biosystems) and snapshots were analyzed using a custom NIS‐Elements macro (Nikon, Minato; Kleczko et al., 2018). Cyst size, index, and number were all calculated automatically and measurements were made using this program. The cystic index (percent of the cross‐sectional area that was cystic), the number of cysts per cross‐sectional area, and cystic area in μm^2^ were determined separately in the cortex and medulla in each rat on three transverse sections (mid‐pole, upper, and lower poles) in both left and right kidney and two sections of the liver. Tissue tears that could be identified at ×40 magnification were excluded from the analysis.

### 2.7 Immunohistochemistry protocol

Tissue sections were deparaffinized and rehydrated, then incubated in sodium citrate buffer (pH 6.0) for 25 min at 100°C to unmask the antigen as previously described (Holditch et al., 2019). Sections were kept in a water bath and cooled to room temperature. Then, the sections were immersed in 3% hydrogen peroxide for 10 min and rinsed in deionized water for 5 min to block endogenous peroxidase activity. Blocking was performed using Vectastain® Elite® ABC Kit blocking serum for 30 min at room temperature. Primary antibodies were diluted in Tris‐buffered saline with Tween20 (TBST; PCNA antibody # 13110 from Cell Signaling Technology) and incubated overnight in a humidified room at 4°C. Immunoreactions were detected using the Vectastain® standard protocol with 3,3′‐diaminobenzidine tetrahydrochloride hydrate (DAB) counterstained with hematoxylin. Dehydrated slides were analyzed for DAB‐positive staining using macros provided by Aperio ImageScope.

### 2.8 Quantification of immunohistochemistry staining

Positive staining cells were counted using Aperio ImageScope (Leica Biosystems) by an operator blinded to the treatment modality. The pathology slide viewing software in Aperio ImageScope enables extensive image analysis including length measurements and counting of positive staining cells. Tubules less than 50 μm in diameter were defined as noncystic tubules. For the noncystic areas, 15–20 fields of view at X40 magnification per sample were randomly selected for noncystic quantification. Positive staining was counted in cysts with a diameter of ~75–200 μm to avoid potential changes in the tubular epithelium covering massive cysts, as well as sensitivity and selection artifacts between noncystic and dilated, possibly precystic tubules. For histological analysis, 50–75 medullary cysts per tissue section were randomly selected for histological analysis.

### 2.9 Metformin serum levels

Samples (10 μL) were analyzed by liquid chromatography with tandem mass spectrometry (LC/MS–MS). Metformin and d6‐metformin were purchased from Toronto Research Chemicals. Control serum was purchased from BioIVT. An Applied Biosystems Sciex 4000 (Applied Biosystems) equipped with a Shimadzu HPLC (Shimadzu Scientific Instruments, Inc.) and a Shimadzu auto‐sampler (Nexera SIL‐30ACMP UHPLC 6‐MTP cooled auto‐sampler) were used. Liquid chromatography employed an Agilent Technologies, Zorbax extended‐C18 250 × 4.6 mm, 5‐micron column equipped with a C18 column guard at 40°C with a flow rate of 0.4 mL/min.

The mobile phase consisted of A: 10 mM (NH_4_OAc), 0.1% formic acid in H_2_O and B: (1:1) acetonitrile:ethanol. The chromatography method used was 5% B for 0.5 min, then ramped to 95% B at 2.5 min, and held for 4.5 min. Next, it was ramped back to 5% B at 8.0 min and held for 2.0 min for a 10.0 min total run time. *Metformin* and *d6‐metformin* (internal standard) were monitored via electro‐spray ionization positive ion mode (ESI+) using the following conditions: (i) an ion‐spray voltage of 5500 V; (ii) temperature, 450°C; (iii) curtain gas (CUR; set at 20) and Collisionally Activated Dissociation (CAD; set at 12) gas were nitrogen; (iv) Ion Source gas one (GS1) and two (GS2) were set at 35 and 50, respectively; (v) entrance potential was set at 10 V; (vi) quadruple one (Q1) and (Q3) were set on low resolution; (vii) dwell time was set at 200 ms; and (viii) de‐clustering potential (DP), collision energy (CE), and collision cell exit potential (CXP) are voltages (V). Samples (10 mL) were analyzed by LC/MS–MS. Settings: *Metformin* (*t*_R_ = 6.6 min) 130.04 → *m/z* 70.89 (quantitative MRM), DP = 46, CE = 29, CXP = 6; 130.04 → 59.88 *m/z* (confirmatory MRM), DP = 46, CE = 19, CXP = 4; *d6‐Metformin* (*t*_R_ = 6.6 min) 136.08 → 76.92 *m/z* (Internal Standard MRM), DP = 31, CE = 31, CXP = 8. A standard curve (0.63–1291 ng/mL; *R* ^2^ = 0.9999) using control serum was prepared in triplicate, frozen (−80°C) subjected to one freeze–thaw cycle. For every 100 mL of serum, 200 mL of extraction solution (1:1 methanol:acetonitrile‐containing *d6‐metformin*) was added, vortex mixed (5 s), and then centrifuged at 11,000 rpm for 5.0 min. The supernatants were transferred to a 96‐well plate and analyzed by LC/MS–MS.

### 2.10 Statistical analysis

Using the G power calculator (Faul et al., 2007), to detect a 50% decrease in cyst index or cyst count, the effect size *d* is 1.75, alpha error 0.05, power 0.8, and the sample size per group is 6. Student's *t* test was used for comparisons between two independent groups. Values were expressed as mean ± SEM, and a *p* value of <0.05 was considered statistically significant.

## 3 RESULTS

### 3.1 MET (300 mg/kg) resulted in weight loss, had no effect on cyst indices, worsened kidney function, and increased serum l‐lactate levels

**FIGURE 1:**
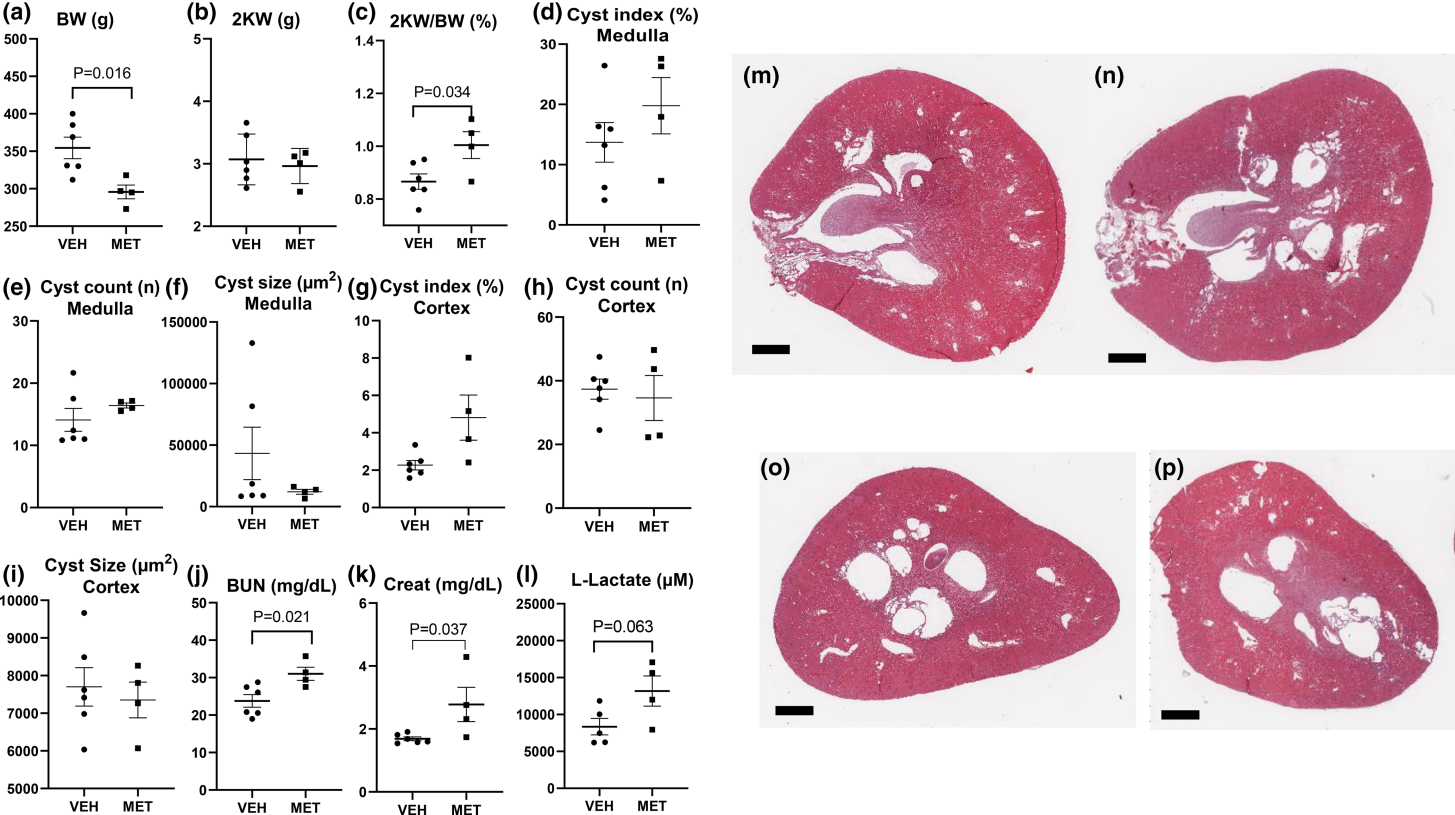
Metformin (MET; 300 mg/kg) resulted in weight loss, had no effect on cyst indices, worsened kidney function, and increased l‐lactate levels. The effect of vehicle (VEH) vs. MET 300 mg/kg on body weight (a), two kidney weights (b), kidney weight corrected for body weight (c), cyst index (the cross‐sectional area of the kidney that was cystic) (d), cyst number per cross‐sectional area (e), cyst size (f) in the medulla, cyst index (g), cyst number per the cross‐sectional area (h), cyst size (i) in the cortex, BUN (j), serum creatinine (k), and serum lactate levels (l). Representative images of hematoxylin–eosin sections from the mid‐pole of the kidney in vehicle‐ (m) and MET‐treated kidneys (n). Representative images from the upper pole of the kidney in vehicle‐ (o) and MET‐treated kidneys (p). Scale bar = 1000 μm. Comparisons between two groups were made using Student's *t* test. A *p* value of <0.05 was considered statistically significant. Values are expressed as the mean ± SEM. BW, total body weight; 2KW, two kidney weight; 2KW/BW (%), 2 kidney weight to body weight ratio expressed as a percentage; creat = serum creatinine.

MET 300 mg/kg resulted in 17% weight loss (Figure 1a) and had no effect on two kidney weights (Figure 1b) and increased kidney weight corrected for body weight, most likely due to reduced body weight (Figure 1c). Two rats in the MET 300 mg/kg group died. MET had no effect on cyst index (the cross‐sectional area of the kidney that was cystic; Figure 1d), cyst number (Figure 1e), or cyst size (Figure 1f) in the medulla. MET had no effect on cyst index (Figure 1g), cyst number (Figure 1h), and cyst size (Figure 1i) in the cortex. MET 300 mg/kg resulted in an increase in BUN (Figure 1j), serum creatinine (Figure 1k), and serum lactate levels (Figure 1l). Representative images of vehicle and MET‐treated kidneys showing similar cyst indices in sections from the mid‐pole of the kidney are shown in Figure 1m,n and in sections from the upper pole of the kidney are shown in Figure 1o,p.

### 3.2 MET (300 mg/kg) had no effect on heart weight or liver cysts

**FIGURE 2:**
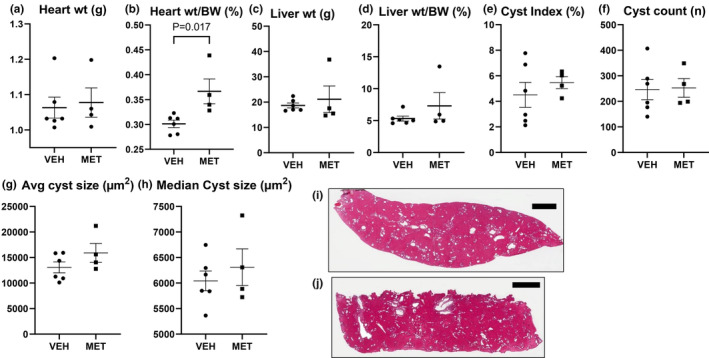
Metformin (MET; 300 mg/kg) had no effect on heart weight or liver cysts. Heart weight (a), heart weight corrected for body weight (b), liver weight (c), and liver weight corrected for body weight (d) in vehicle (VEH) and MET. Liver cyst index (% of the liver) (e), liver cyst count (number of cysts) (f), average (g), and median liver cyst size (h) are shown. Cyst index, count, and size data for two separate sections of each liver are shown. Representative images of liver cysts are shown in (i and j). Scale bar = 2 mm. Comparisons between the two groups were made using Student's *t* test. A *p* value of <0.05 was considered statistically significant. Values are expressed as the mean ± SEM. wt, weight; BW, body weight.

Heart weight was not increased in male PCK versus wild‐type controls. Heart weight to body weight ratio (%) was 0.31 ± 0.02 in +/+ rats (*N* = 6) vs. 0.31 ± 0.02 in PCK rats (*N* = 6) (*p* value = not significant). MET had no effect on heart weight (Figure 2a). MET resulted in an increase in the heart weight to body weight ratio (Figure 2b) most likely due to the loss of body weight (Figure 1a).

MET had no effect on liver weight (Figure 2c) or liver weight corrected for body weight (Figure 2d). MET had no effect on liver cyst index (Figure 2e), liver cyst number (Figure 2f), or liver cyst size (Figure 2g,h). Representative images of liver cysts are shown in Figure 2i,j. MET 300 mg/kg/day orally, but not 150 mg/kg/day orally, has been shown to decrease liver cyst index in PCK rats (Sato et al., 2021). In that study, male PCK rats were treated with MET 300 mg/kg/day orally for 11 weeks from week 6 until 17 weeks of age (Sato et al., 2021). In the present study, where MET had no effect on liver cysts, PCK rats had milder liver disease as they were treated at an earlier age for 9 weeks from week 4 to week 12 of age when the liver cystic disease was less severe.

### 3.3 MET increases p‐S6 (mTORC1), but has no consistent effect in the kidneys on p‐AMPK^T172^ , p‐ACC^S79^ , LC3‐II, p62, p‐Akt^T308^, or p‐Akt^S473^

**FIGURE 3:**
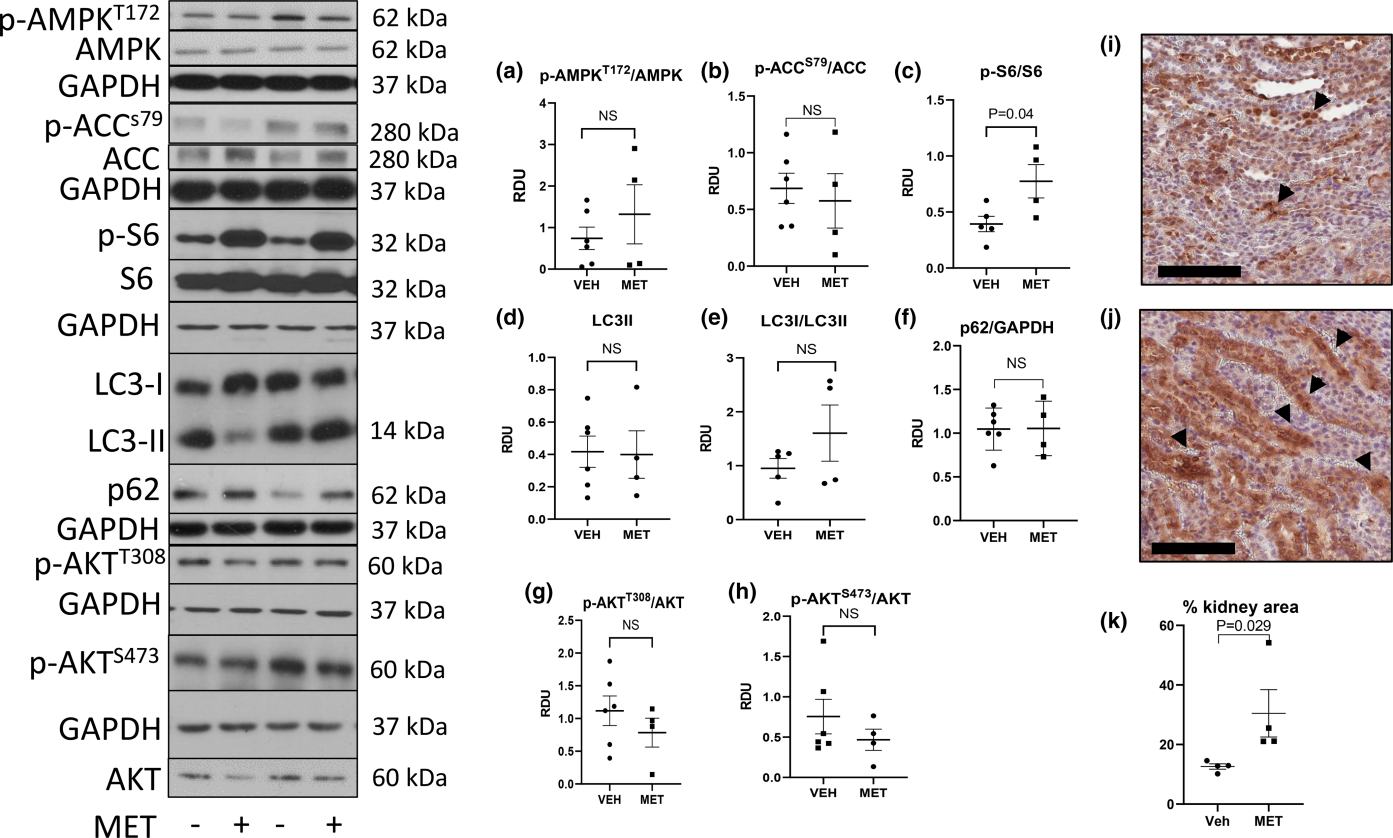
Metformin (MET) has no effect on p‐AMPK^T172^ or p‐ACC^S79^, increases p‐S6 (mTORC1), but has no consistent effect on LC3‐II (autophagy), p‐Akt^T308^ (upstream of mTORC1), or p‐Akt^S473^ (mTORC2). Quantitative immunoblot analysis for mTOR proteins in vehicle‐ (VEH) and MET‐treated kidneys. Representative densitometry of immunoblot analysis of proteins in the kidney is demonstrated: p‐AMPK (a), p‐ACC (b), p‐S6 (c). LC3‐II (d), the conversion of LC3‐I to LC3‐II (e), p62 (f). p‐Akt^T308^ (g), p‐Akt^S473^ (h). Immunohistochemistry staining for p‐S6 (brown) mostly in tubules in vehicle‐ (i) and metformin (j)‐treated PCK rats. Scale bar = 100 μm. Quantitation of p‐S6 staining is shown in (k). Comparisons between the two groups were made using Student's *t* test. A *p* value of <0.05 was considered statistically significant. Values are expressed as the mean ± SEM. NS, not significant; RDU, relative densitometry units.

AMP‐activated protein kinase (AMPK) plays a key role in the regulation of energy homeostasis (Hardie et al., 2016). AMPK activation regulates the metabolism, protein synthesis, and cell growth through the inhibition of the TSC2/mTOR pathway (Hardie et al., 2016). The premise of the study was that activated p‐AMPK would suppress p‐S6, a marker of mTORC1. AMPK is a sensitive metabolic sensor and phosphorylation may be sensitive to quick changes in metabolism during and after removal of kidneys (Leonhard et al., 2019). AMPK phosphorylates acetyl‐CoA carboxylase (ACC) resulting in ACC inhibition that leads to increased rates of myocardial fatty acid oxidation (FAO) and ATP production (Hardie et al., 2016). p‐ACC^S79^ is an accepted marker of AMPK activation. So, to confirm the lack of effect of metformin on p‐AMPK, we also measured p‐ACC. Metformin had no effect on p‐AMPK (Figure 3a) or p‐ACC (Figure 3b) and unexpectedly resulted in an increase in p‐S6 (Figure 3c).

Microtubule‐associated proteins 1A/1B light chain 3B, also known as LC3, is a central protein in the autophagy pathway and the most widely used marker of autophagosomes. The lipid‐modified form of LC3, called LC3‐II, plays a role in autophagosome membrane expansion and autophagosome lysosome fusion events. p62 also known as Sequestosome 1 (SQSTM1, p62) is a ubiquitin‐binding protein that binds the autophagosomal membrane protein LC3 and brings p62‐containing protein aggregates to the autophagosome (12). Lysosomal degradation of autophagosomes leads to a decrease in p62 during active autophagy while autophagy inhibitors increase p62. MET has been shown to activate autophagy in some cases by decreasing mTORC1 (Lu et al., 2021). MET had no effect on the absolute levels of LC3‐II (Figure 3d) or the conversion of LC3‐I to LC3‐II (Figure 3e), a marker of ongoing autophagy or p62, a marker of lysosomal degradation (Figure 3f).

p‐Akt^T308^ is a protein kinase that is activated by insulin and various growth and survival factors. AKT plays a critical role in regulating cell growth by phosphorylating and inactivating tuberin (TSC2), an inhibitor of mTOR or directly phosphorylating mTOR in a rapamycin‐sensitive complex containing raptor (Dan et al., 2002). Phosphorylation of AKT at the serine 473 (S473) residue is a target of the mTOR complex 2 (mTORC2). It has been shown that AMPK can directly activate mTORC2 (Kazyken et al., 2019). MET had no effect on p‐Akt^T308^ (Figure 3g) or p‐Akt^s473^ (Figure 3h).

The increase in p‐S6 with MET seen on immunoblot analysis was confirmed by immunohistochemistry. On immunohistochemistry staining there was increased p‐S6 mostly in tubules in vehicle‐treated (Figure 3i) vs. MET‐treated (Figure 3j) PCK rats. Quantitation of increased staining is shown in Figure 3k. While, in most studies, AMPK suppresses mTORC1 (Lu et al., 2021), it has been described that MET increases mTORC1 signaling in embryonic pancreas cells (Dan et al., 2002) and in hypothalamic tissue (Kazyken et al., 2019) suggesting that tissue responses of p‐S6 to MET can be both tissue and developmental stage specific (Kugita et al., 2017).

Original immunoblots with molecular weight markings are shown in Figure S1.

### 3.4 MET (300 mg/kg) had no effect on proliferation

**FIGURE 4:**
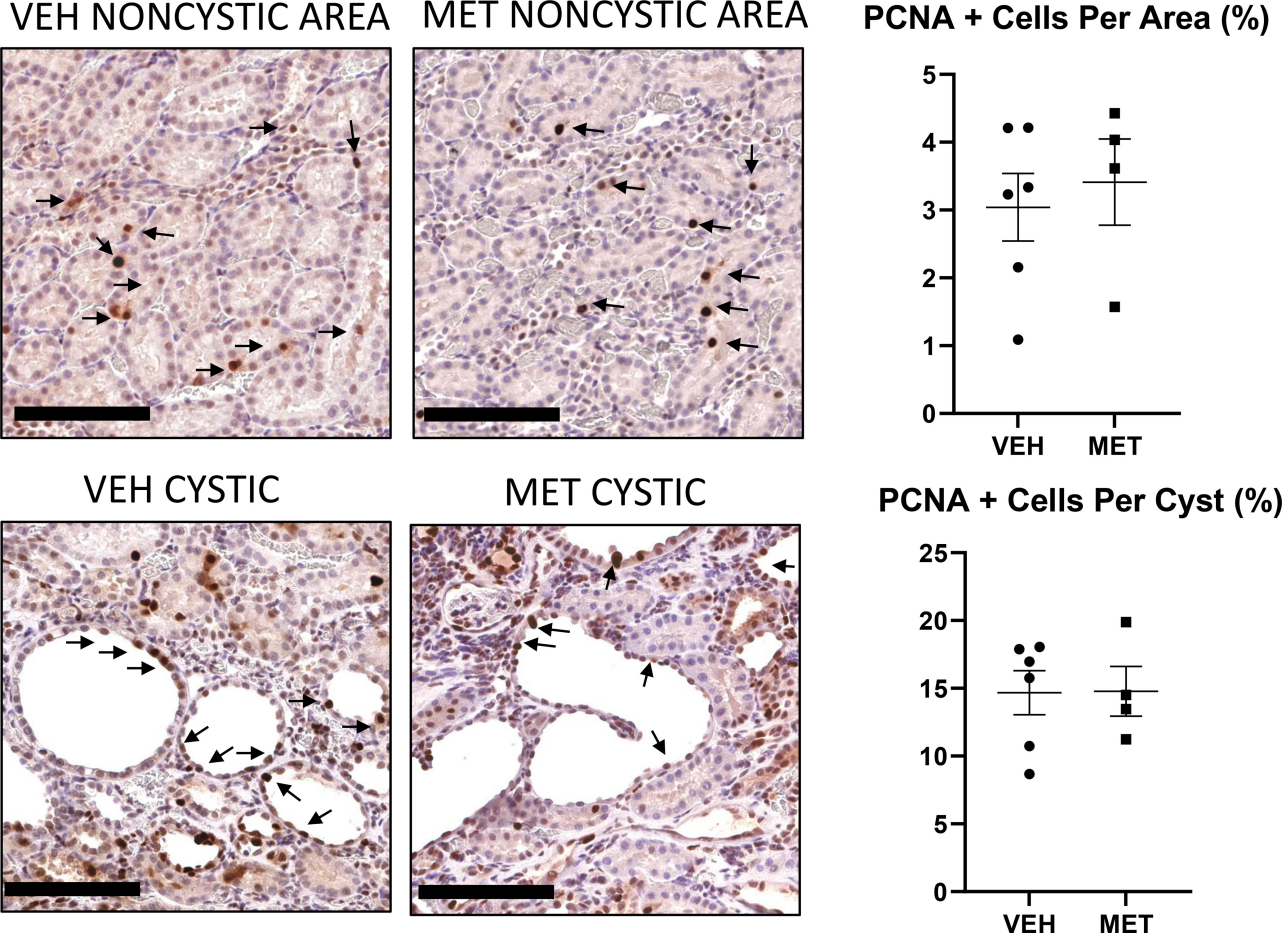
Metformin (MET; 300 mg/kg) had no effect on proliferation. The effect of vehicle (VEH) versus MET on PCNA staining (arrows) in the noncystic tubules or cystic tubular cells in the PCK rats. Scale bar = 100 μm. Comparisons between the two groups were made using Student's *t* test. A *p* value of <0.05 was considered statistically significant. Values are expressed as the mean ± SEM.

The premise of the study was that MET would activate AMPK, block mTORC1 and inhibit proliferation. As MET had no effect on AMPK (Figure 3a) and increased p‐S6 (mTORC1; Figure 3c), we did not expect to see an effect of MET on proliferation. MET had no effect on proliferation, as indicated by PCNA staining in either the noncystic tubules or cystic tubular cells in the PCK rats (Figure 4).

### 3.5 MET (150 mg/kg) had no effect on cyst indices, kidney function, or serum l‐lactate levels

**FIGURE 5:**
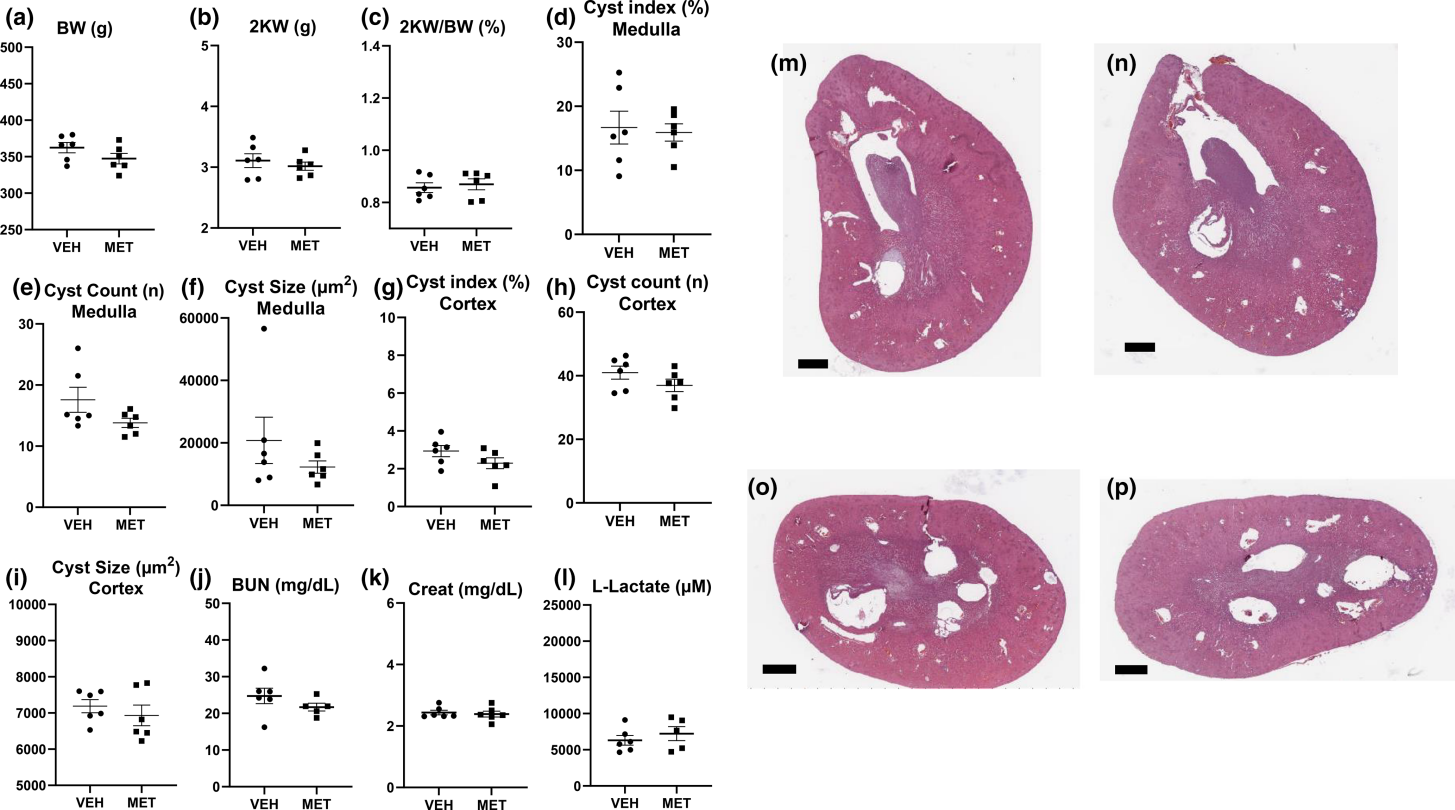
Metformin (MET; 150 mg/kg) had no effect on cyst indices, kidney function, or serum l‐lactate levels. The effect of vehicle (VEH) versus MET 150 mg/kg on body weight (a), kidney weight (b), kidney weight corrected for body weight (c), cyst index (the cross‐sectional area of the kidney that was cystic) (d), cyst number per cross‐sectional area (e), cyst size (f) in the medulla, cyst index (g), cyst number per cross‐sectional area (h), cyst size (i) in the cortex, BUN (j), serum creatinine (k), and serum lactate levels (l). Representative images of hematoxylin–eosin sections from the mid‐pole of the kidney in vehicle‐ (m) and MET‐treated kidneys (n). Representative images from the upper pole of the kidney in vehicle (o) and MET‐treated kidneys (p). Scale bar = 1000 μm. Comparisons between the two groups were made using Student's *t* test. A *p* value of <0.05 was considered statistically significant. Values are expressed as the mean ± SEM. BW = total body weight, 2KW = two kidney weight, 2 K/BW (%) = 2 kidney weight to body weight ratio expressed as a percentage, creat = serum creatinine.

MET 150 mg/kg had no effect on body weight (Figure 5a), kidney weight (Figure 5b), and kidney weight corrected for body weight (Figure 5c). MET 150 mg/kg had no effect on cyst index (the cross‐sectional area of the kidney that was cystic; Figure 5d), cyst number (Figure 5e), and cyst size (Figure 5f) in the medulla. MET had no effect on cyst index (Figure 5g), cyst number (Figure 5h), and cyst size (Figure 5i) in the cortex. MET 150 mg/kg had no effect on BUN (Figure 5j), serum creatinine (Figure 5k), and serum lactate levels (Figure 5l). Representative images of vehicle and MET‐treated kidneys showing similar cyst indices in sections from the mid‐pole of the kidney are shown in Figure 5m,n and in sections from the upper pole of the kidney are shown in Figure 5o,p.

### 3.6 MET (150 mg/kg) had no effect on liver or heart weight

**FIGURE 6:**
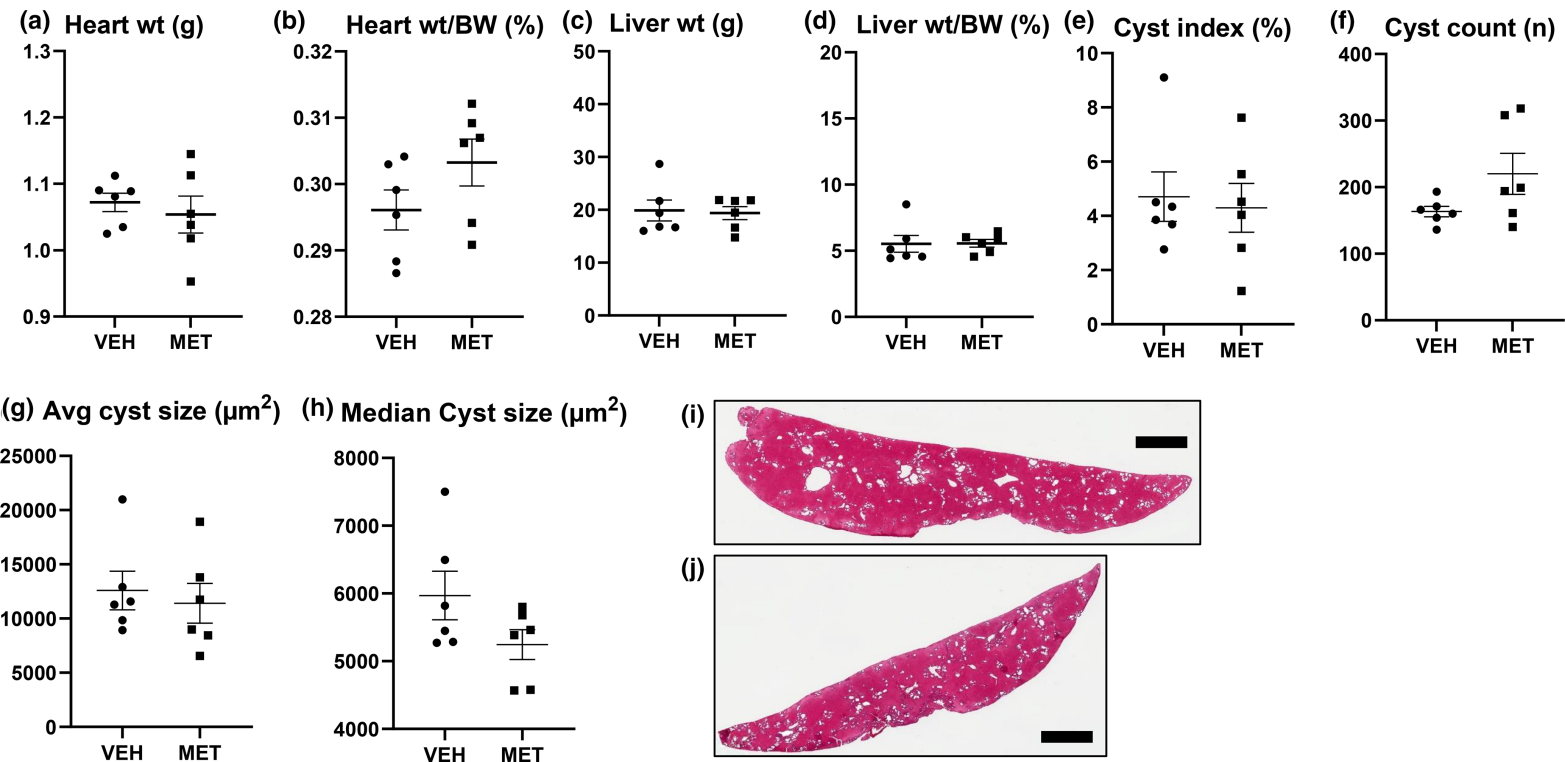
Metformin (MET; 150 mg/kg) had no effect on liver or heart weight. Heart weight (a), heart weight corrected for body weight (b), liver weight (c), and liver weight corrected for body weight (d) in vehicle (VEH) and MET (Met). Liver cyst index (% of the liver) (e), cyst count (number of cysts) (f), average (g), and median cyst size (h) is shown. Representative images of liver cysts in vehicle‐ (i) and metformin‐treated (j) rats. Scale bar = 2 mm. Cyst index, count, and size data for two separate sections of each liver are shown. Comparisons between the two groups were made using Student's *t* test. A *p* value of <0.05 was considered statistically significant. Values are expressed as the mean ± SEM. wt, weight; BW, body weight.

MET had no effect on heart weight (Figure 6a) or heart weight corrected for body weight (Figure 6b). MET had no effect on liver weight (Figure 6c) or liver weight corrected for body weight (Figure 6d). MET had no effect on liver cyst index (Figure 6e), liver cyst number (Figure 6f), or liver cyst size (Figure 6g,h). Representative images of liver cysts are shown in Figure 6i,j.

### 3.7 MET (150 mg/kg) had no effect on p‐AMPK, p‐S6 (mTORC1), autophagy proteins, or p‐Akt

**FIGURE 7:**
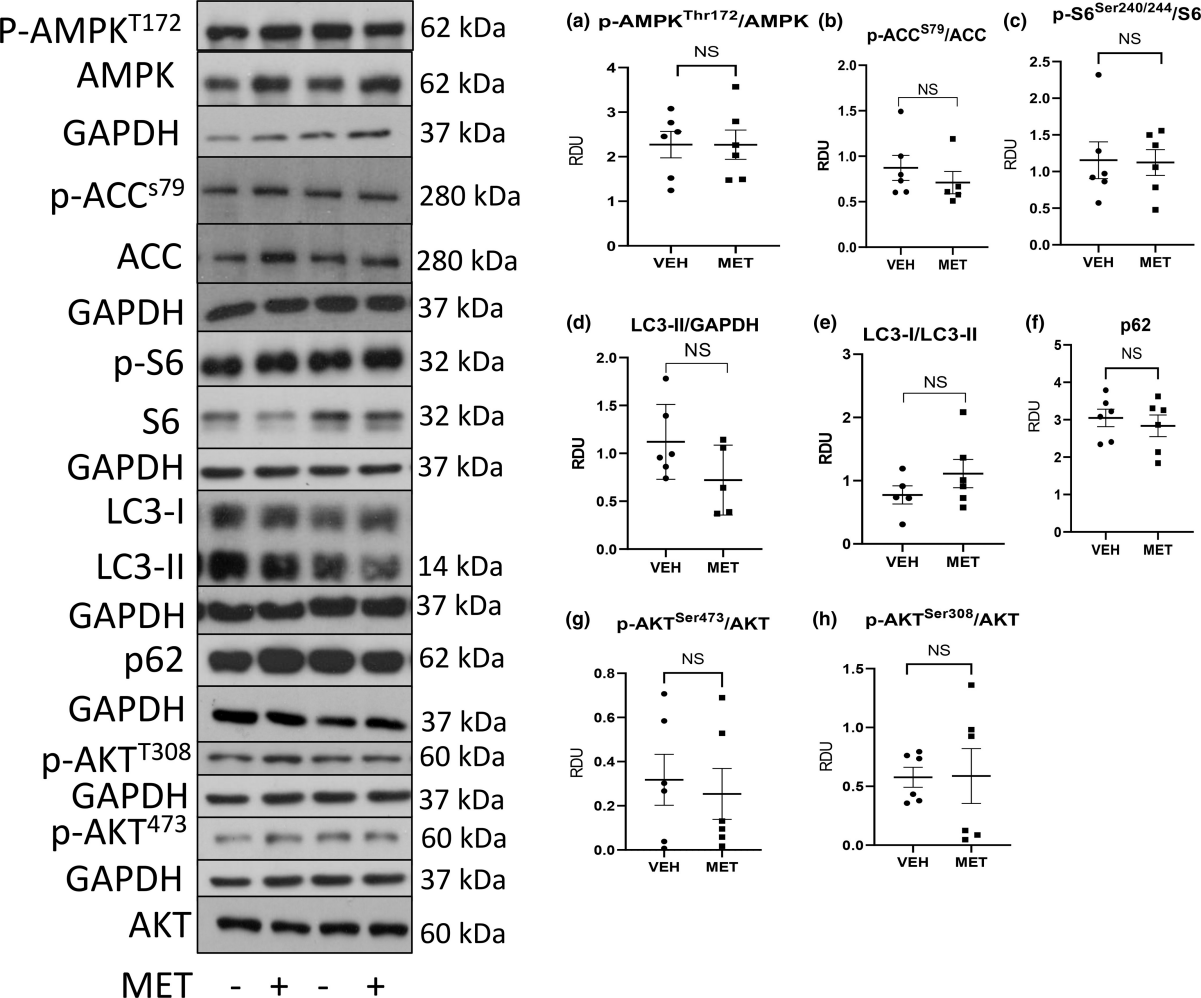
Metformin (MET; 150 mg/kg) has no effect on p‐AMPK, p‐ACC, p‐S6 (mTORC1), autophagy (LC‐3, p62), p‐Akt^T308^, or p‐Akt^s473^ (mTORC2). Quantitative immunoblot analysis for mTOR proteins in vehicle‐ (VEH) and MET‐treated kidneys. Representative densitometry of immunoblot analysis of proteins in the kidney is demonstrated. p‐AMPK (a), p‐ACC (b), p‐S6 (c), LC3‐II (d), the conversion of LC3‐I to LC3‐II (e), p62 (f), p‐Akt^S473^ (g), and p‐Akt^T308^ (h). Comparisons between the two groups were made using Student's *t* test. A *p* value of <0.05 was considered statistically significant. Values are expressed as the mean ± SEM. NS, not significant; RDU, relative densitometry units.

Unlike the higher dose of MET that increased p‐S6 (Figure 3c), MET 150 mg/kg had no effect on p‐AMPK (Figure 7a), p‐ACC (Figure 7b), and p‐S6 (Figure 7c), the autophagy proteins LC3‐II (Figure 7d,e), p62 (Figure 7f), pAKT^s473^ (Figure 7g), or pAKT^T308^ (Figure 7h).

Original immunoblots with molecular weight markings are shown in Figure S1.

### 3.8 MET (150 mg/kg) had no effect on proliferation

**FIGURE 8:**
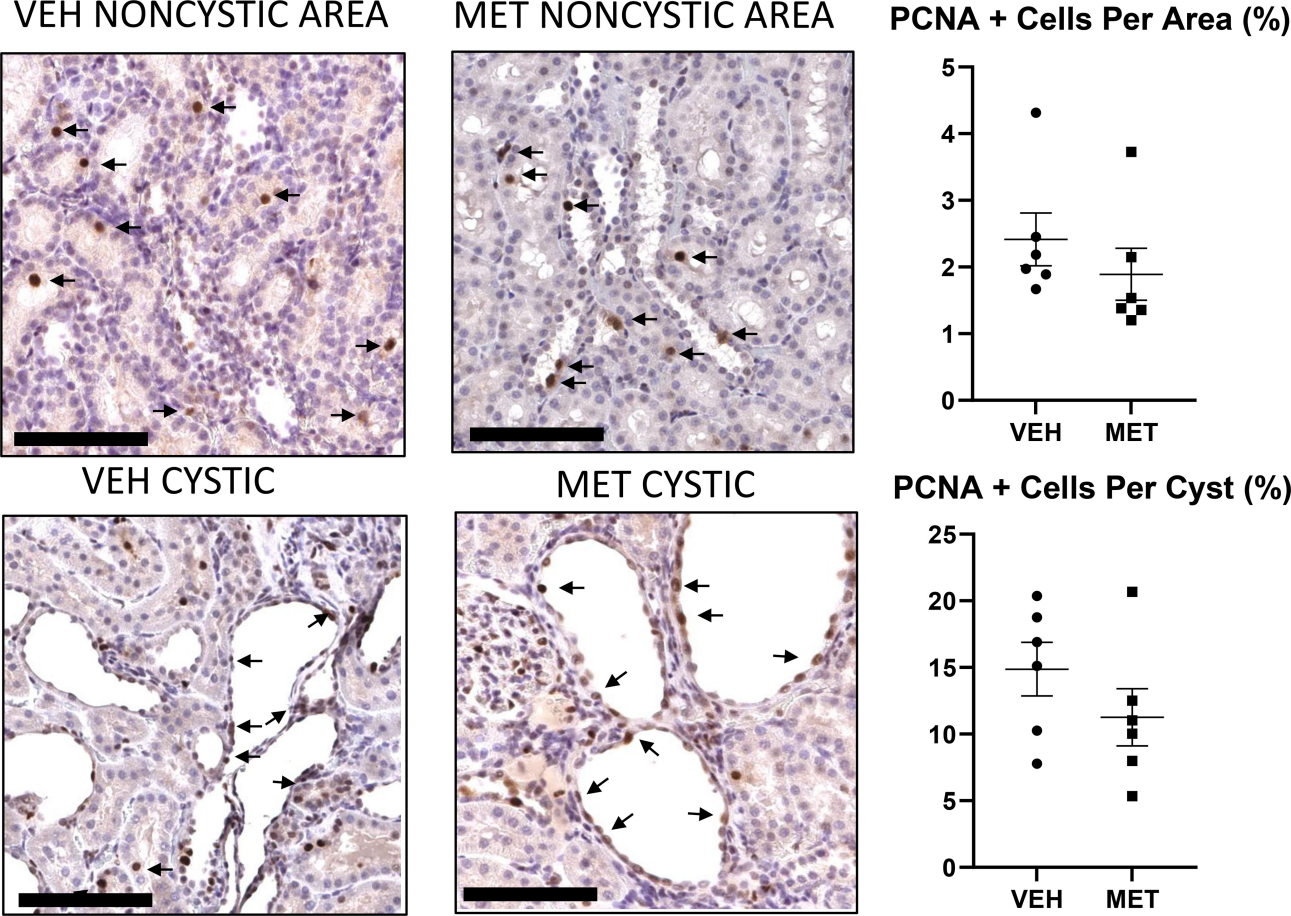
Metformin (MET; 150 mg/kg) had no effect on proliferation. The effect of vehicle (VEH) versus MET on PCNA staining (arrows) in the noncystic tubules or cystic tubular cells in the PCK rats. Scale bar = 100 μm. Comparisons between two groups were made using Student's *t* test. A *p* value of <0.05 was considered statistically significant. Values are expressed as the mean ± SEM.

The premise of the study was that MET would activate AMPK, block mTORC1, and inhibit proliferation. MET had no effect on proliferation, as indicated by PCNA staining in either the noncystic tubules or cystic tubular cells in the PCK rats (Figure 8).

### 3.9 Serum MET levels

Peak MET levels were obtained at the end of the study, 2 h after the last dose of MET. In rats treated with MET 300 mg/kg IP, the mean serum MET level (μmol/L) was 359 ± 137 (*N* = 4). In rats treated with MET 150 mg/kg IP, the mean serum MET level (μmol/L) was 32 ± 11 (*N* = 6). Thus, doubling the dose of MET resulted in a 10‐fold increase in mean serum MET levels and toxicity suggesting that the dosage range between therapeutic and toxic is narrow.

The therapeutic concentration for MET is not well defined but has been described to range from 0.1 to 20.7 μmol/L (Sutkowska et al., 2021), 6–30 μmol/L in type 2 diabetic patients (Chang et al., 2022), or 10–40 μmol/L in rodents (He & Wondisford, 2015). Serum levels of ~5–10 μmol/L are similar to that seen in patients treated with the usual clinical dose (i.e., 1.5–2.5 g/day; Leonhard et al., 2019). In the 150 mg/kg ‐treated rats, mean peak MET levels were 32 μmol/L in a steady state after 8 weeks of treatment, and trough levels are predicted to be about 50% lower than peak levels (Li et al., 2020) which would still be in the therapeutic range.

## 4 DISCUSSION

In the present study, MET 300 mg/kg IP at an early stage of the disease (days 28–84 of age) resulted in MET blood levels more than 10 times the therapeutic level, weight loss, and nephrotoxicity and had no effect on PKD in rats. Although MET 150–300 mg/kg orally are the standard doses used in rats, toxic effects on the kidney are seldom measured. The present study suggested that 300 mg/kg IP may be too high a dose to use in rats and that renal toxicity may mask a therapeutic effect on cyst growth and kidney function in PKD. Thus, we used a lower dose of 150 mg/kg which is below the NOAEL of 200 mg/kg/day orally in Sprague Dawley rats (Quaile et al., 2010). The lower dose of MET did not affect renal function or serum lactate levels, but this lower dose still did not have a therapeutic effect, despite therapeutic blood levels. The lactate accumulation and nephrotoxicity at a 300 mg/kg dose but not at a 150 mg/kg dose suggests that the safe dosage range may be small.

MET is not metabolized in the body and is eliminated in the urine with a half‐life of 5 h. However, MET is a known substrate for various drug transporters in the body including organic cation transporters (OCTs), plasma membrane monoamine transporter (PMAT), and multidrug toxin extrusions (MATEs) transporters (Yang et al., 2021). Hepatic OCT1, intestinal OCT3, renal OCT2 on the tubule basolateral membrane, and MATE1 on hepatocytes and MATE1/2‐K on tubule apical membrane control MET elimination (Yang et al., 2021). It is possible that doubling the dose of MET caused MATE transporters that eliminate MET to become saturated resulting in steeply elevated blood levels. For example, MATE1 is located at the apical membrane of the hepatocyte and MATE1/2‐K are located in the kidneys. If higher concentrations of MET decrease hepatic secretion and/or decrease tubular secretion, this might help to explain the steeply elevated MET plasma levels.

Preclinical and clinical studies of MET in PKD have shown mixed results. MET reduced cyst growth in vitro and in ex vivo kidney cyst models and in vivo in two rapid cyst growth models of PKD (Takiar et al., 2011). Salsalate, a direct AMPK activator, but not MET reduced cyst growth and improved kidney function in an adult onset slowly progressive model of PKD more analogous to human disease than the rapid models (Leonhard et al., 2019). In the Pkd1^RC/RC^ hypomorphic gene model of moderately progressive PKD in the 129S/C57BL‐6J background, MET from 3 to 9 months of age reduced cyst growth, reduced blood pressure and improved kidney function (Pastor‐Soler et al., 2022). However, in the Pkd1^RC/RC^ model of slowly progressive PKD in the C57BL/6J background, MET at an earlier stage of the disease from days 35 to 64 of age had no effect on cyst growth (Chang et al., 2022). In Pkd1 miRNA transgenic mice, MET resulted in faster cyst growth, worse renal function and lactate accumulation (Chang et al., 2022). In the same Pkd1 miRNA transgenic model, a lower dose of MET had no effect on cyst growth (Chang et al., 2022). In the present study in PCK rats, a therapeutic dose of MET had no effect on cyst growth or kidney function. In a prospective randomized controlled double‐blind feasibility study, 51 adults with ADPKD were treated with MET (maximum dose 2000 mg/d) or placebo for 12 months. 50% of the MET‐treated participants completed the treatment phase on the full dose compared with 100% in the placebo group. In exploratory analyses, changes in height‐adjusted total kidney volume (htTKV) or eGFR were not significantly different between the groups (Brosnahan et al., 2022). In a randomized trial of MET in ADPKD patients (TAME PKD), the annual change of GFR was −1.71 with MET versus 3.07 with placebo, but the decrease did not reach statistical significance (Perrone et al., 2021). Based on the TAME PKD study results, it was suggested that a larger clinical trial be performed.

Next, we considered whether the variable effects of MET on cyst growth in preclinical studies in mice may have been related to different dosing or the timing of treatment. In a rapid severe Pkd 1 knockout mouse model, MET for less than 8 days slowed cyst growth (Takiar et al., 2011). In a slowly progressive Ksp‐Pkd1del conditional knockout (KO) model in mice, MET resulted in serum levels equivalent to that in diabetic patients but did not have a therapeutic effect on PKD (Leonhard et al., 2019). In the moderately progressive Pkd1^RC/RC^ mouse model in the 129S/C57BL‐6J background, MET in the later stages of the disease improved disease parameters (Pastor‐Soler et al., 2022). However, in the slowly progressive male Pkd1^RC/RC^ mouse model in the C57BL‐6J background, MET treatment at an early stage of the disease resulted in increased lactate levels and lack of a therapeutic effect on cyst growth (Chang et al., 2022). In pkd1 miRNA mice treated with short‐term MET for approximately 4 weeks, with blood levels equivalent to the therapeutic levels in humans, there was worse PKD and increased plasma lactate levels (Chang et al., 2022). Thus, in four published studies in mice and the current study in rats, MET was tested in seven rodent models at similar doses of 150–300 mg/kg. MET reduced cyst growth in two rapid severe models in Pkd1 −/− mice (Takiar et al., 2011) and Pkd1^RC/RC^ mice treated at an advanced stage (Pastor‐Soler et al., 2022). MET did not reduce cyst growth in PCK rats at an early stage (present study) nor in slowly progressive PKD murine models treated at an early stage (Chang et al., 2022; Leonhard et al., 2019). In fact, MET increased cyst growth and was toxic when administered at a later stage of the disease in Pkd1 miRNA mice (Chang et al., 2022). These studies overall suggest that MET may slow cyst growth at more advanced stages of the disease, but it can also be toxic. Thus, there is a lack of efficacy in the majority of preclinical studies and in the first randomized trials in PKD patients. The efficacy at more advanced stages of disease and the potential for toxicity at both the early and late stages of the disease are discouraging for any future clinical trials of MET in PKD patients.

AMPK, a supersensitive intracellular energy sensor, is an attractive target in PKD (Padovano et al., 2018). First, the cystic fibrosis transmembrane conductivity regulator (CFTR) that increases fluid secretion into cyst lumens is inhibited by AMPK‐mediated phosphorylation of the chloride channel (Hallows et al., 2003). Second, AMPK increases the GTPase‐activating protein (GAP) activity of the TSC1/2 tuberous sclerosis complex by phosphorylating the AMPK tuberin protein (TSC2) resulting in mTORC1 inhibition (Kim et al., 2011). AMPK can also paradoxically increase mTOR (Kazyken et al., 2019; Sarbassov et al., 2005). However, MET can have beneficial effects in ADPKD independent of AMPK. It can inhibit cellular cAMP production which causes cyst growth by inhibiting adenylate cyclase activity (Miller et al., 2013) or reduce excessive aerobic glycolysis (“Warburg effect”) that is thought to play a role in cyst growth (Rowe et al., 2013). So, next, we considered the evidence that MET activates p‐AMPK in the polycystic kidney. Part of the rationale for using MET in PKD is that it can activate AMPK and suppress mTORC1‐driven proliferation of cells lining the cysts. mTORC1 is an AMPK target and AMPK can both directly and indirectly regulate mTORC1 (Cork et al., 2018). In the present study, both doses of MET were unable to activate AMPK in the polycystic kidney. While MET has been shown to activate AMPK in the kidney in other kidney diseases, for example, renal ischemia (Seo‐Mayer et al., 2011) and diabetic nephropathy (Han et al., 2018), evidence that MET can activate AMPK in the PKD kidney is lacking. In the current study in rats, both doses of MET had no effect on p‐AMPK or p‐ACC in the kidney. The first study that demonstrated a therapeutic effect of MET in mice demonstrated that MET activated AMPK in normal kidneys but did not demonstrate that MET activates AMPK in the polycystic kidneys (Takiar et al., 2011). Two other preclinical studies in mice (Leonhard et al., 2019; Pastor‐Soler et al., 2022) could not demonstrate activation in AMPK in the kidney, thought to be due to technical difficulties and long‐term exposure to the drug resulting in compensatory signaling pathways. In fact, the study of MET 150 mg/kg in Pkd1^RC/RC^ mice showed that MET resulted in a decrease rather than an increase in AMPK (Chang et al., 2022). In a mouse model of diabetic nephropathy, there was a large decrease in p‐AMPK in the kidney, and activation of AMPK with MET had therapeutic effects (Han et al., 2021). We have previously demonstrated a three‐fold increase in AMPK in Pkd1^RC/RC^ kidneys (Atwood, Brown, et al., 2020) suggesting that AMPK may be maximally activated in PKD kidneys and that MET is unable to activate AMPK further in PKD kidneys. Also, the three‐fold increase in AMPK in Pkd1^RC/RC^ kidneys was not able to suppress mTORC1 signaling (Atwood, Brown, et al., 2020). Thus, it is possible that AMPK activation works best in models of decreased p‐AMPK in the kidney. It is unknown whether MET can activate AMPK in human PKD kidneys. Interestingly, MET (41 mg/kg) is able to activate AMPK and slow cyst growth in a miniature pig model of ADPKD (Lian et al., 2019). Interestingly, in a mouse model expressing a constitutively active mutant of AMPK, there was early onset polycystic kidney phenotype and increased cAMP levels and ERK activation (Wilson et al., 2021). Thus, the maximal activation of p‐AMPK in PKD kidneys may be insufficient to suppress mTORC1 and may even cause cystogenesis in mouse and rat models of PKD (Wilson et al., 2021). To inform further studies of MET in rodents and humans, elucidating the pattern of AMPK activation in the PKD kidney would be important.

MET is used in PKD with the premise that it reduces proliferation of the cells lining the cysts. Next, it was considered whether the usual doses of MET have the capacity to reduce the proliferation of the cystic epithelium, one of the mechanisms of cyst growth. Most in vitro assays revealed that millimolar (mM) levels of MET are required to have antiproliferative effects (Chandel et al., 2016). In cystic epithelial cells, millimolar concentrations of MET were required to have an effect on proliferation (Takiar et al., 2011). Diabetic patients receiving usual doses of MET, 1.5–2.5 g/day, have plasma levels in the 10 μM range. This implies that typical doses of MET in patients may not have an antiproliferative effect in the kidney (Graham et al., 2011). In the two preclinical studies of MET in mice, blood levels were in the 10 μmol/L range, and there was no effect on proliferation and cyst growth (Chang et al., 2022; Leonhard et al., 2019). It is possible that the typical dose of MET achieves blood levels that do not suppress proliferation in the PKD kidneys. However, MET is a cation and is predicted to accumulate 100‐ to 500‐fold in the mitochondria due to the membrane potential (Rena et al., 2017). Thus, micromolar concentrations of MET in the plasma may result in considerably higher concentrations in the mitochondria of cells and have an effect on metabolism independently of AMPK activation. Further preclinical studies of pharmacokinetics and mechanism of action of MET in PKD are needed to guide the design of future human studies.

Next, we considered reasons why MET 300 mg/kg/day resulted in increased BUN and creatinine and 17% weight loss. The worsening kidney function was not associated with increased cyst growth. The lower dose of MET that did not raise lactate levels was not associated with weight loss or worse kidney function suggesting that the weight loss and increased creatinine were related to increased lactate levels. Lactic acidosis from MET is caused by inhibition of gluconeogenesis by blocking pyruvate carboxylase, which leads to accumulation of lactic acid (Blough et al., 2015). In the present study, weight loss could have been from decreased food intake or the effects of lactate on metabolism. For example, moderate L‐lactate administration, in high‐fat diet obese mice, reduced body weight and obesity‐associated insulin resistance (Cai et al., 2022). It is controversial whether increased lactate is the cause or result of kidney injury. Lactate can both cause AKI (Radovic et al., 2019; Yang et al., 2022) by triggering multiple cell signaling pathways or can accumulate due to AKI (Connelly et al., 2017). In the present study, it cannot be concluded whether increased lactate levels caused the kidney injury or whether the lactate levels increased because of the decreased kidney function. A limitation in interpreting the results of the MET 300 mg/kg/day study was the small sample size because two rats died in the treatment group, likely due to MET toxicity, resulting in an *N* = 4.

In the present study in male PCK rats, we were able to determine the effect of MET on cyst growth in both the kidney and the liver. PCK rats develop liver cysts and have increased liver weight by 21 days of age and markedly enlarged liver by 180 days of age (Lager et al., 2001). In PCK rats, MET 300 mg/day orally from week 5 to 17 of age did not affect the body weight or liver weight but reduced liver cyst formation, cholangiocyte proliferation, and fibrosis around the cyst (Sato et al., 2021). In that study, MET increased the phosphorylation of AMPK and TSC2 and decreased the phosphorylation of mammalian target of rapamycin, S6, ERK, and the expression of CFTR, aquaporin I, transforming growth factor‐β, and type 1 collagen in the liver. In the present study, MET treatment at an earlier stage of the disease, when liver cyst index is mild, had no effect on liver cyst growth.

Cardiac hypertrophy is common in PKD patients. In 4‐week‐old PCK rats, before the onset of hypertension or renal failure, there is reduced vascular density and decreased expression of angiogenic factors (Franchi et al., 2020). In 15‐week‐old PCK rats, systolic and diastolic blood pressures were significantly higher compared with control rats (Kugita et al., 2017). MET has been shown to reduce cardiac hypertrophy (Li et al., 2017). In the present study, MET did not decrease heart weight.

In Pkd1^RC/RC^ mice in the 129S/C57BL‐6J background, female mice had more severe disease and MET improved markers of PKD disease severity, primarily in female mice (Pastor‐Soler et al., 2022). In another study in Pkd1^RC/RC^ mice in the C57BL‐6J background, MET had no effect on PKD in males and females were not studied (Chang et al., 2022). The kidney disease severity in the PCK rat is greater in males than females, while liver cystic disease is similar across sexes (Lager et al., 2001). Thus, a limitation of the current study is that only male PCK rats were studied which may limit the generalizability of the findings as ADPKD is a disease with sexual dimorphism. There may be sex‐dependent effects of MET that were not addressed in this study.

In summary, MET (300 mg/kg) resulted in toxic blood levels, weight loss, increased lactate levels, and nephrotoxicity in male PCK rats. A lower dose of MET 150 mg/kg resulted in therapeutic blood levels, no weight loss, did not reduce kidney weight or cyst indices, and had no effect on kidney function or serum lactate levels in male PCK rats. Both doses had no effect on p‐AMPK, p‐ACC, mTOR, and autophagy proteins. Both doses did not decrease proliferation in the cystic kidney. The present study highlights a number of concerns on the use of MET in PKD: (1) Although the hypothesis in most studies is that MET activates AMPK, suppresses mTORC1, and reduces proliferation, few mouse studies actually show activated p‐AMPK, suppressed mTOR, and reduced proliferation. (2) The dosage of MET required to activate p‐AMPK and suppress mTOR or activate autophagy in human PKD kidneys may be too high which can result in increased lactate levels and nephrotoxicity. (3) Blood and tissue levels of MET may be too low to have an effect on proliferation. (4) MET had no effect on early liver cyst growth. (5) The MET dosage range between therapeutic and toxic may be narrow.

## FUNDING INFORMATION

C.L.E. was funded by a Merit Award grant from the Department of Veteran's Affairs (grant nos. I01BX003803‐05) and a grant from the Zell Family Foundation. O.A.O. was funded by an International Postdoctoral Fellowship Grant from the Scientific and Technological Research Council of Turkey. A.S.L. was funded by the National Institutes of Health (grant nos. 5T32DK007135‐46).

## Supporting information


Figure S1.
