# Supplementary material for: Metformin does not slow cyst growth in the PCK rat model of polycystic kidney disease
Source: Physiol Rep. 2023 Aug 31;11(17):e15776. doi: 10.14814/phy2.15776 (PMC10471794; doi:10.14814/phy2.15776)

Supplementary Figure S1: Original blots with marked molecular weights

Fig 3: p-AMPK


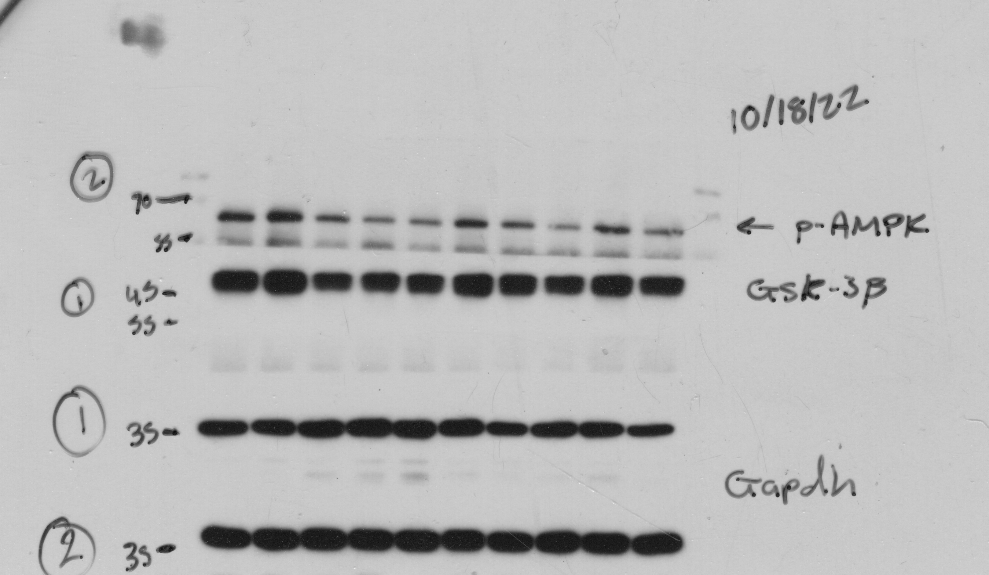


Fig 3: AMPK


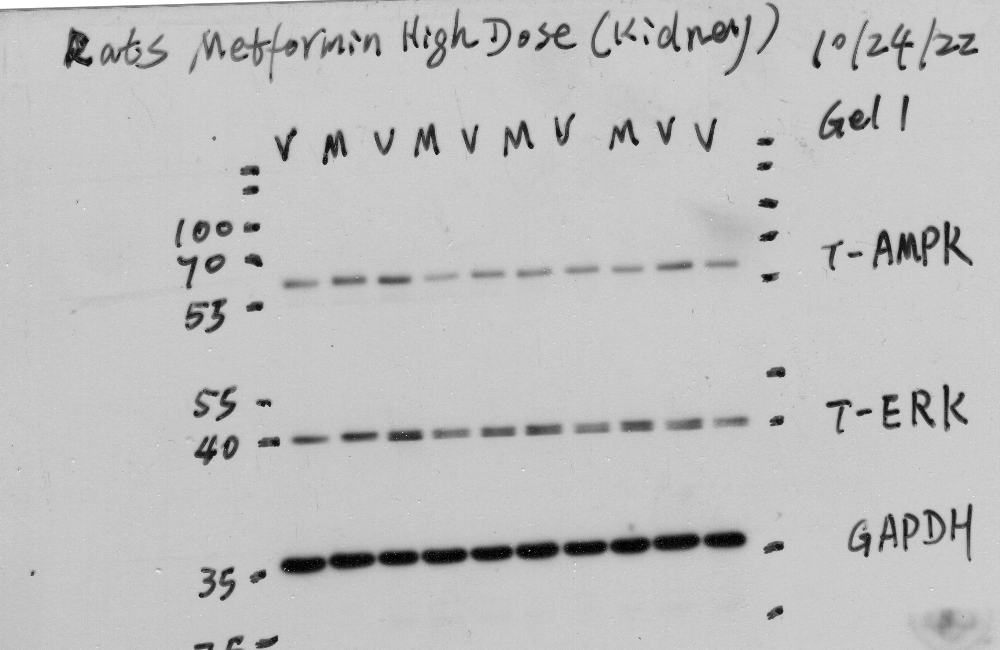


Fig 3: p-ACC and ACC


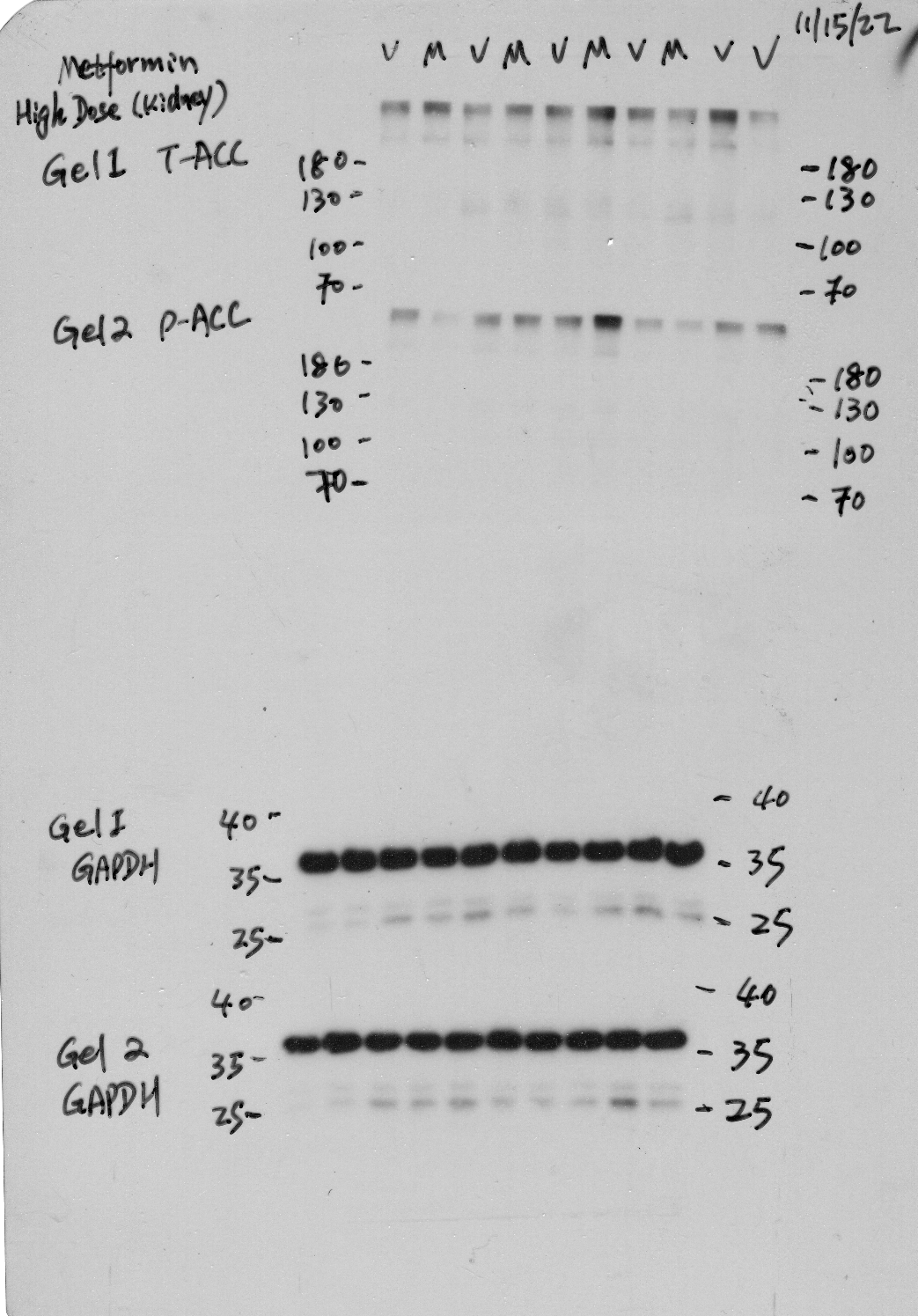


Fig 3: p-S6 and S6


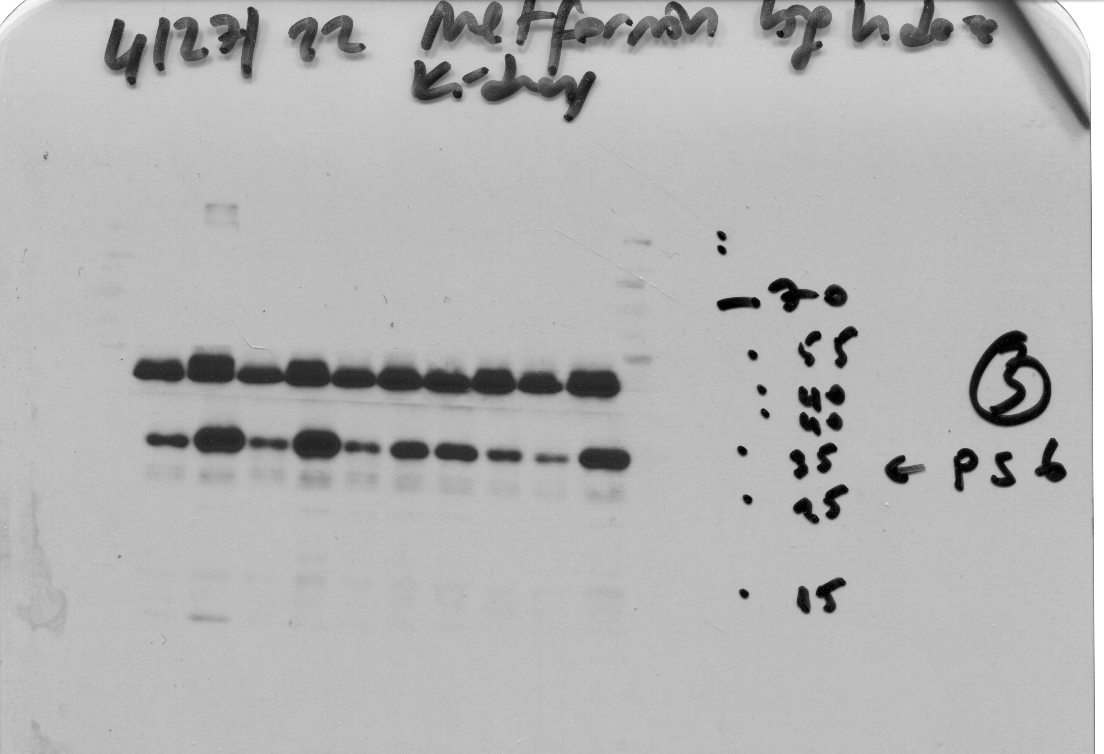


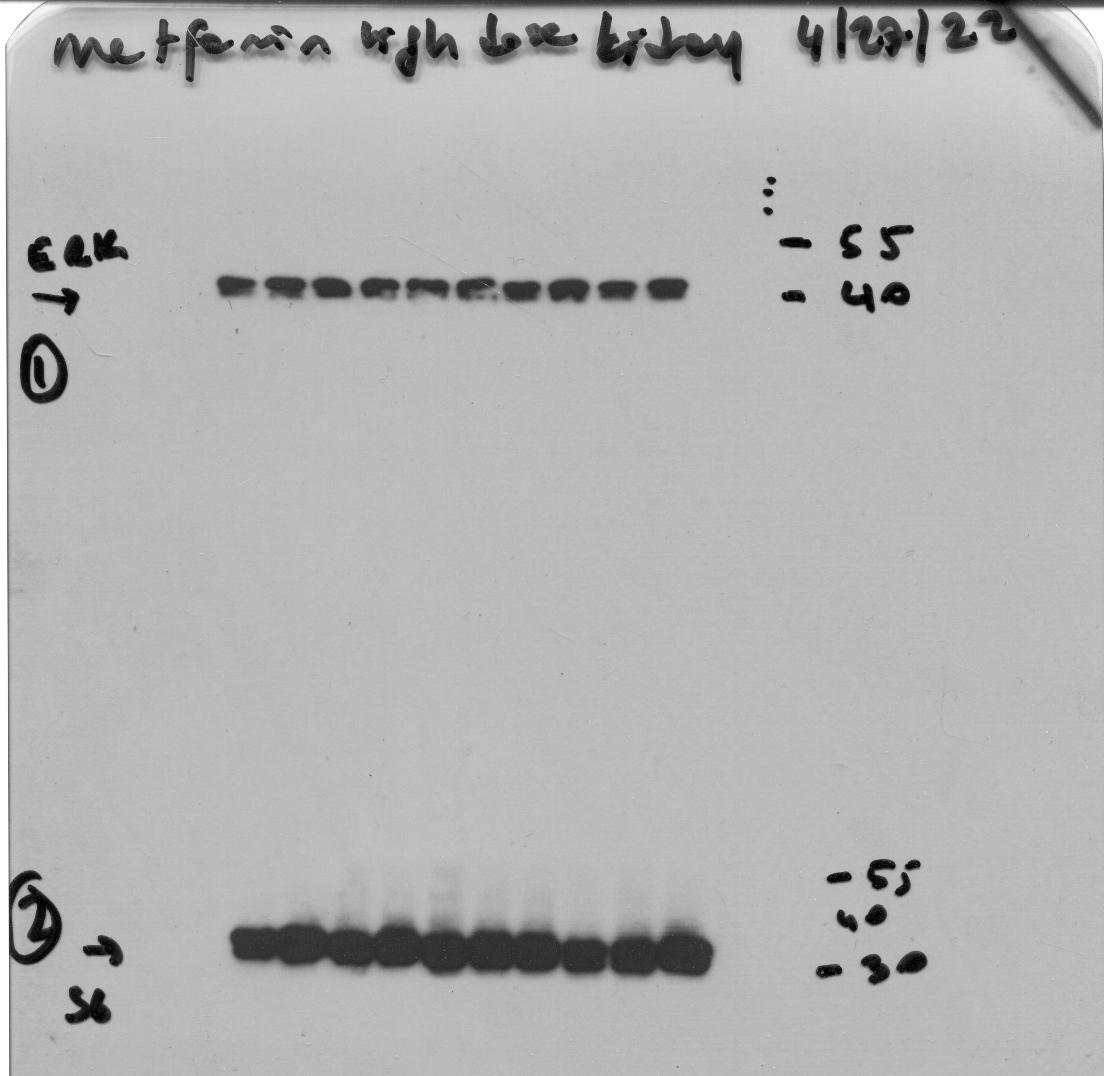


Fig 3: LC3


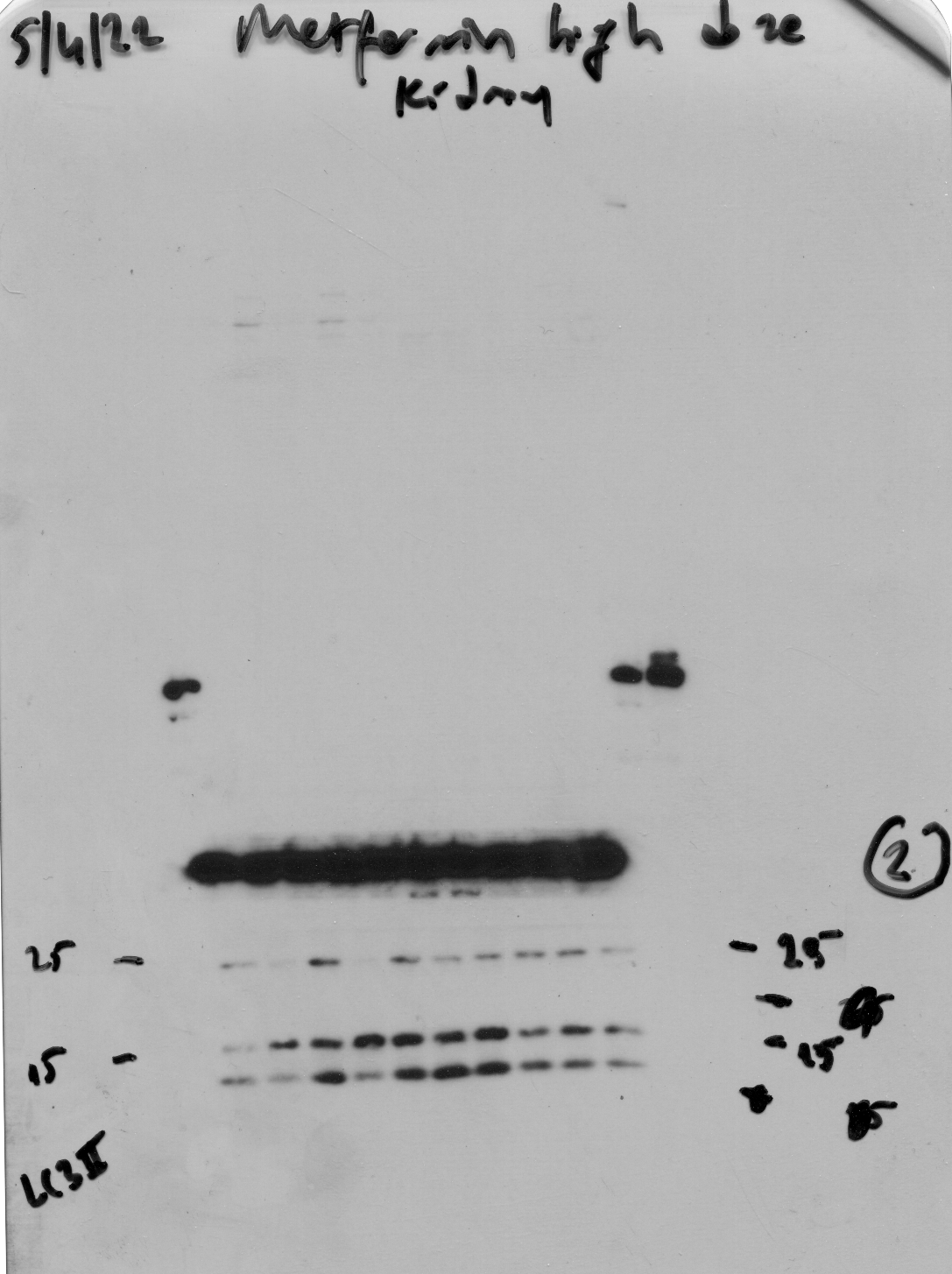


Fig 3: p62


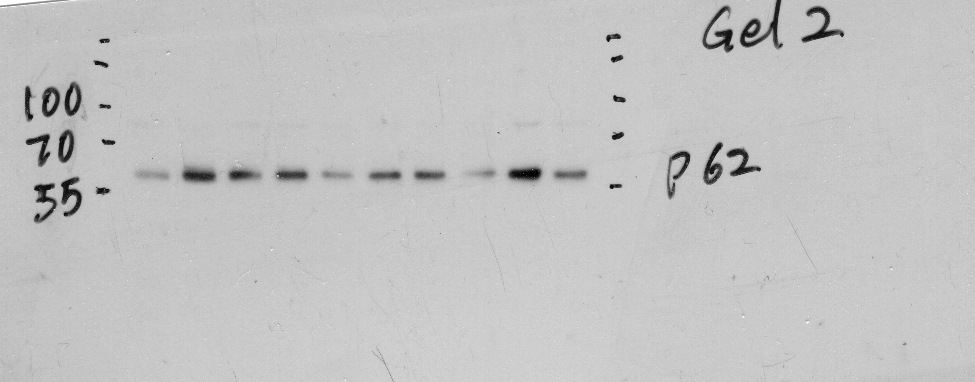


Fig 3. P-AKT^T308^


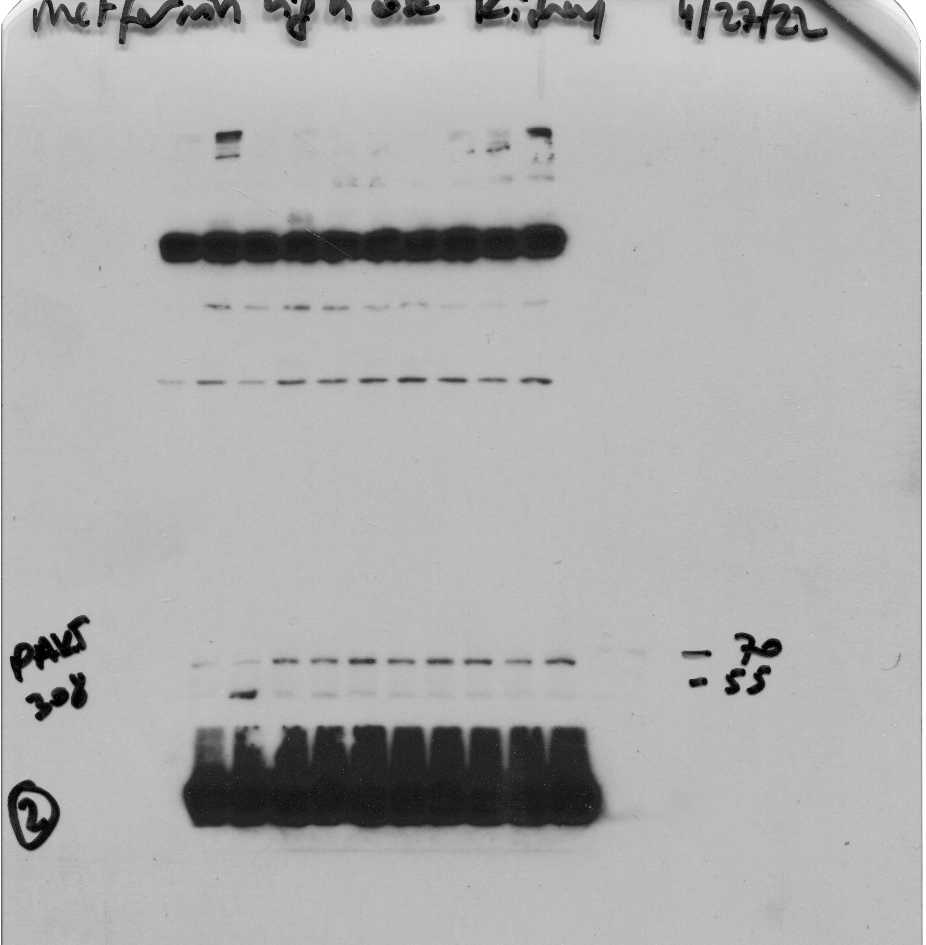


Fig 3: p-Akt^S473^


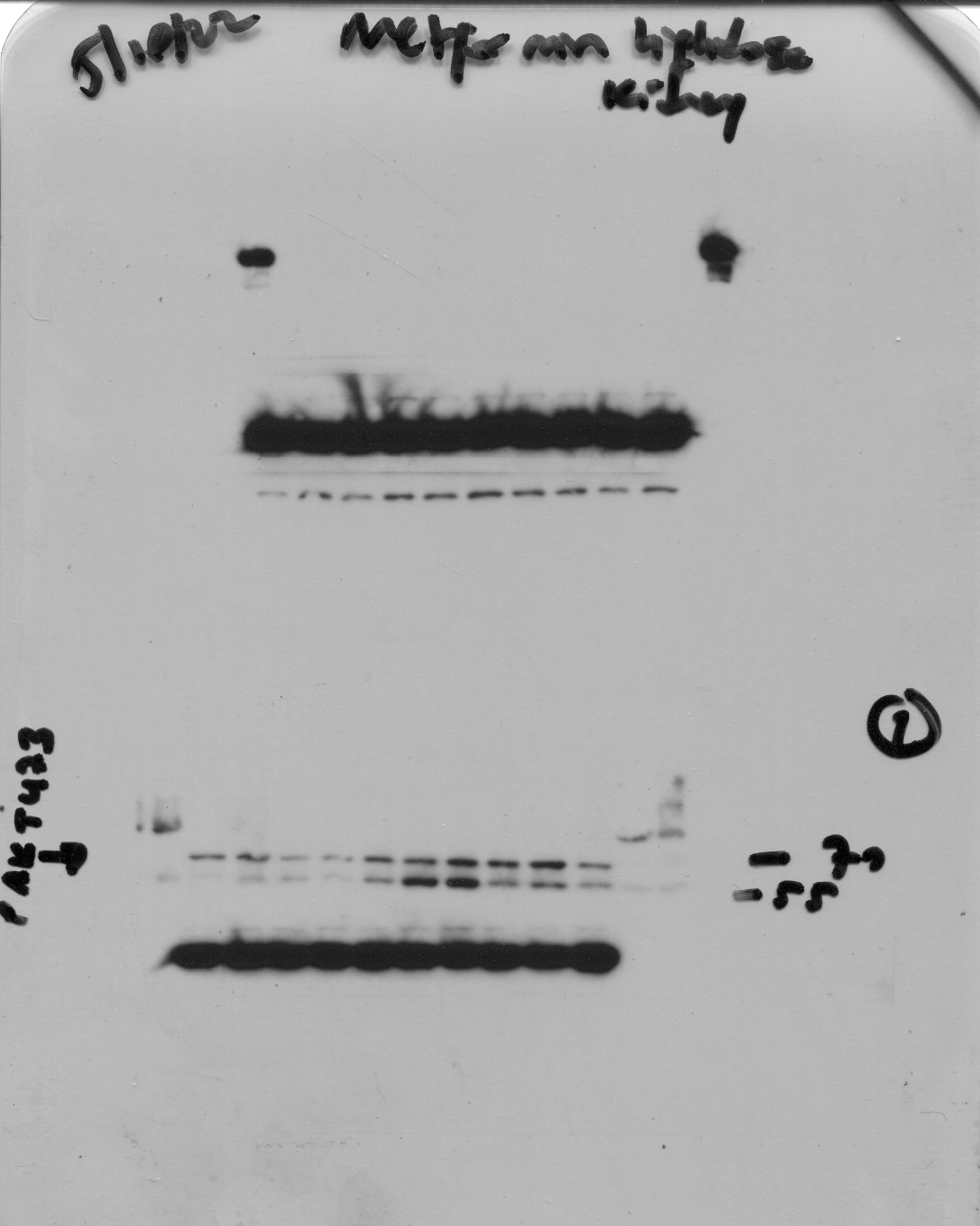


Fig 3. AKT


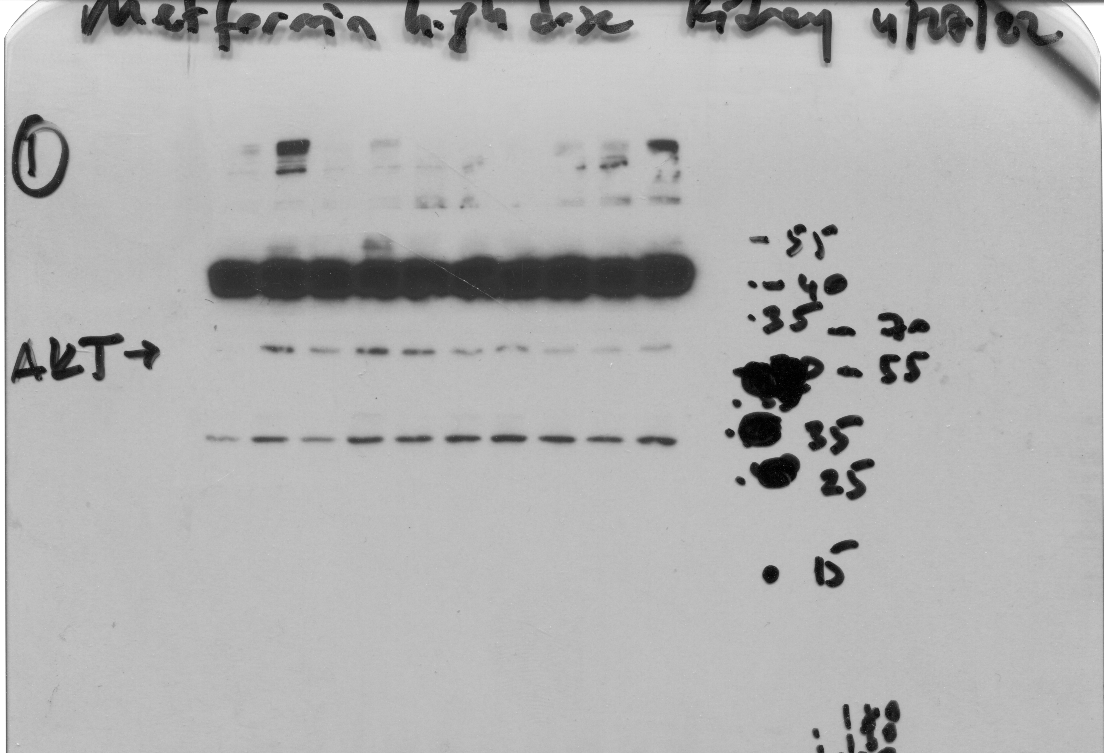


Fig 7: p-AMPK


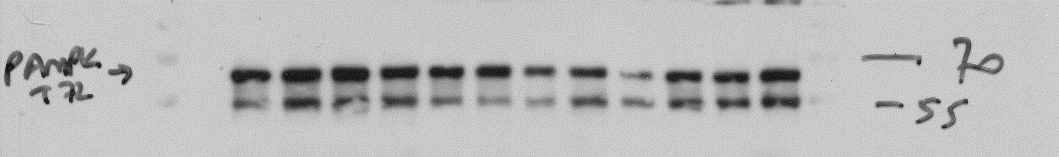


Fig 7: AMPK


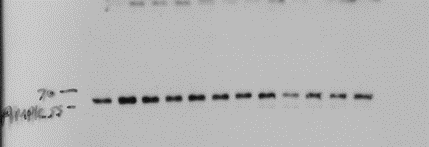


Fig 7: p-ACC and ACC


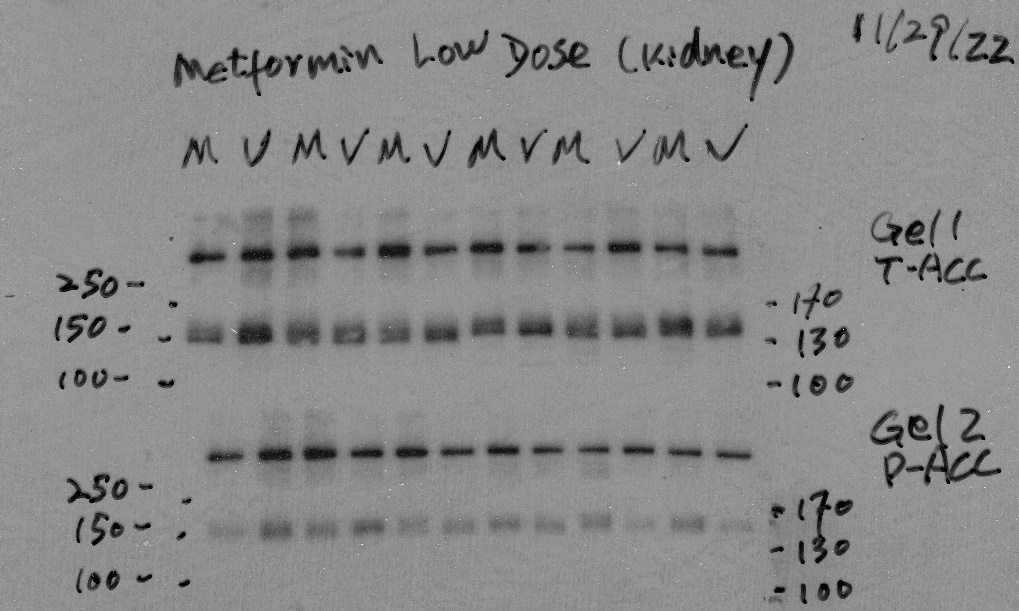


Fig 7: p-S6 and S6


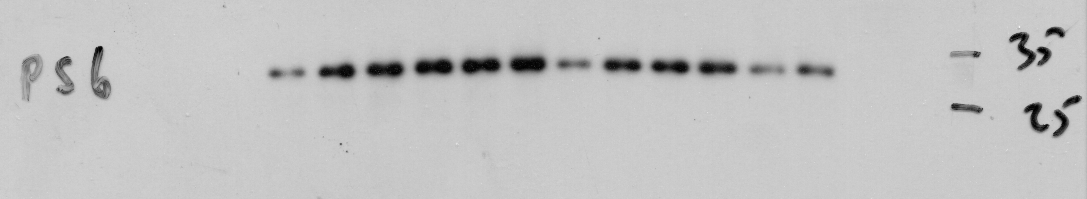


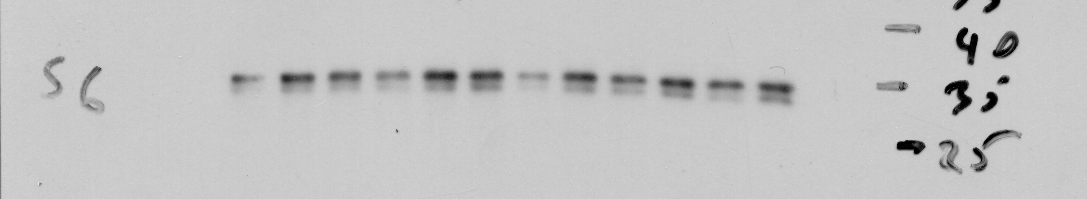


Fig 7: LC3


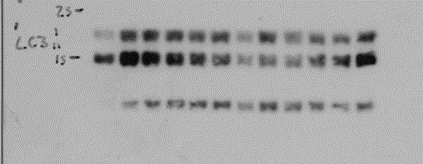


Fig 7: p62


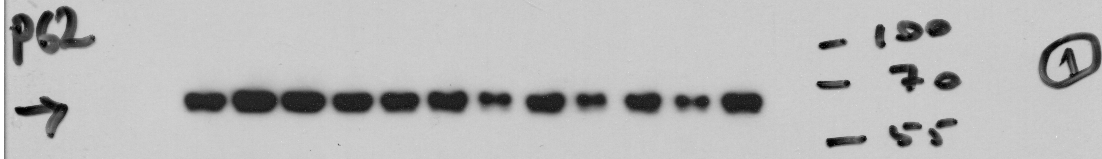


Fig 7: p-AKT^T308^


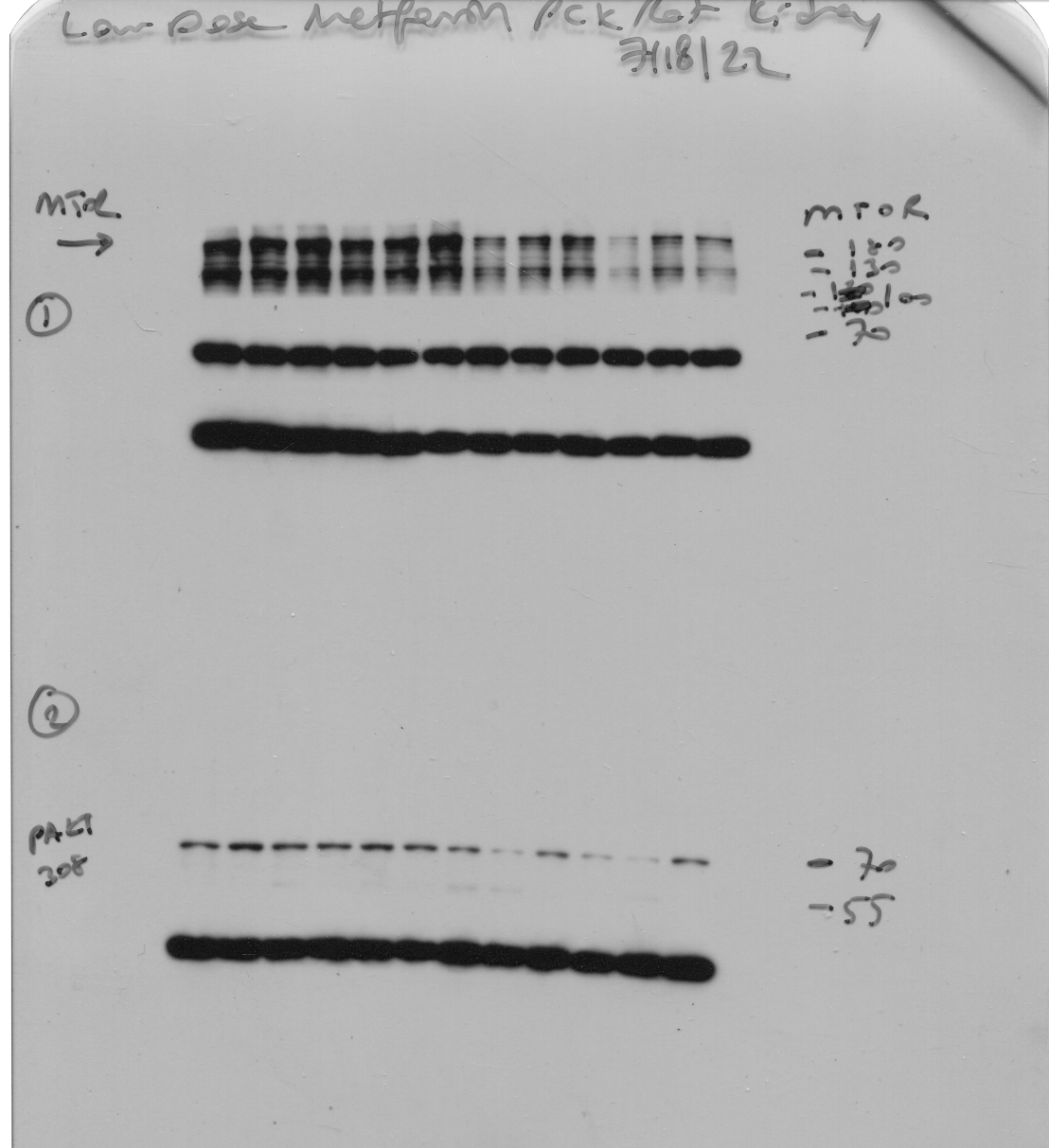


Fig 7. pAKT^S473^


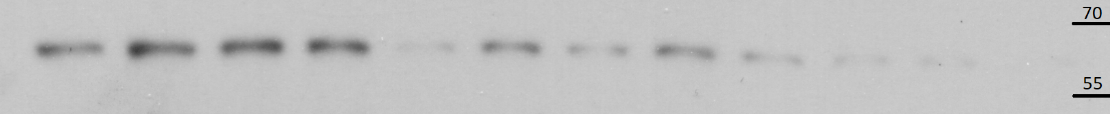


Fig 7: AKT


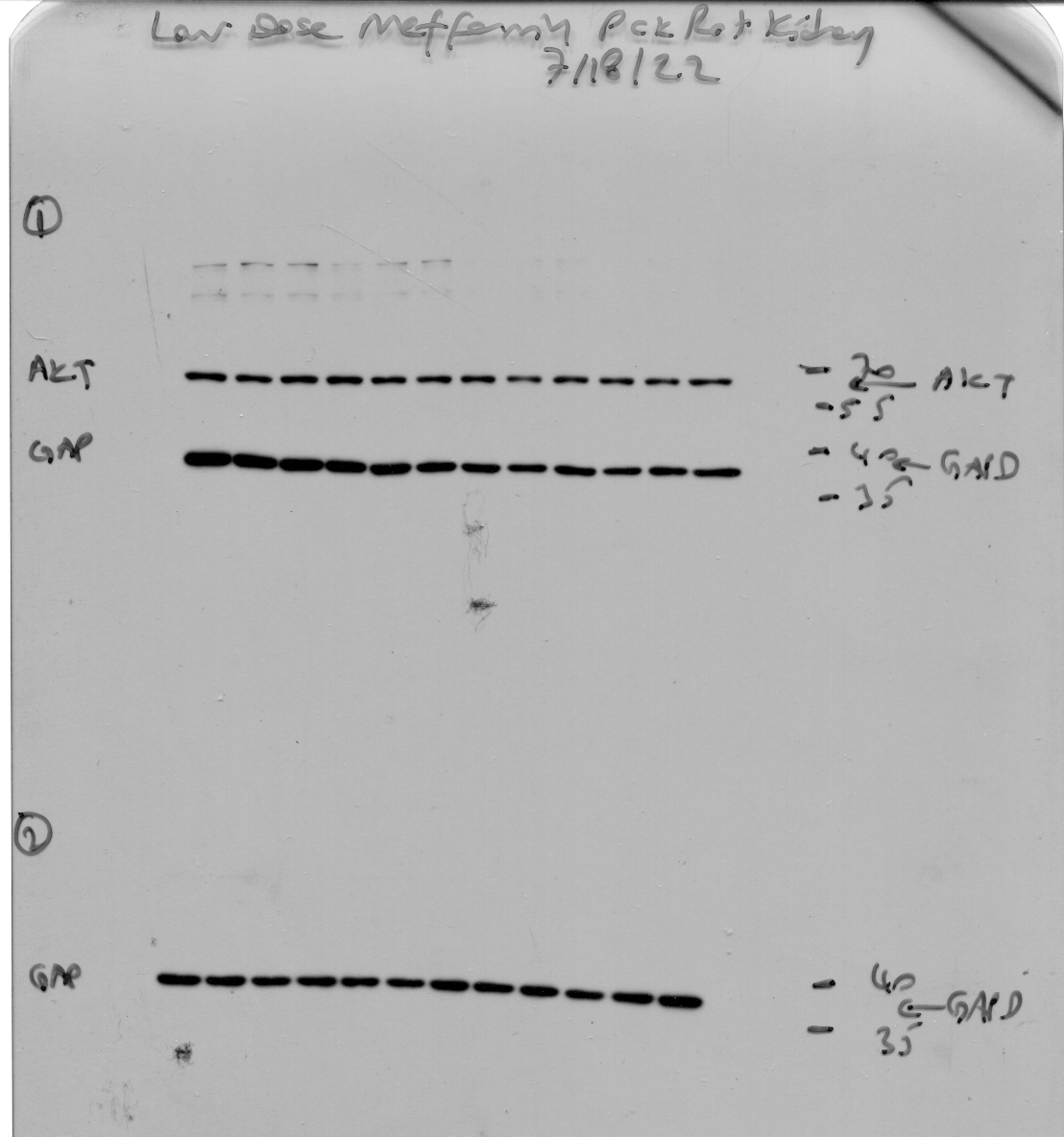

Supplement: Supplementary file 1 — Figure S1. [file PHY2-11-e15776-s001.docx]
